# Interleukin-6 Signaling Drives Fibrosis in Unresolved Inflammation

**DOI:** 10.1016/j.immuni.2013.10.022

**Published:** 2014-01-16

**Authors:** Ceri A. Fielding, Gareth W. Jones, Rachel M. McLoughlin, Louise McLeod, Victoria J. Hammond, Javier Uceda, Anwen S. Williams, Mark Lambie, Thomas L. Foster, Chia-Te Liao, Christopher M. Rice, Claire J. Greenhill, Chantal S. Colmont, Emily Hams, Barbara Coles, Ann Kift-Morgan, Zarabeth Newton, Katherine J. Craig, John D. Williams, Geraint T. Williams, Simon J. Davies, Ian R. Humphreys, Valerie B. O’Donnell, Philip R. Taylor, Brendan J. Jenkins, Nicholas Topley, Simon A. Jones

**Affiliations:** 1 Cardiff Institute of Infection and Immunity, Cardiff University, School of Medicine, Heath Park, Cardiff CF14 4XN, UK; 2 Centre for Innate Immunity and Infectious Diseases, Monash Institute for Medical Research, Monash University, Clayton, VIC 3168, Australia; 3 Department of Nephrology, University Hospital of North Staffordshire and Institute for Science and Technology in Medicine, Keele University, Stoke-on-Trent ST4 7QB, UK; 4 Institute of Nephrology, Institute of Molecular and Experimental Medicine, School of Medicine, Cardiff University, Heath Park, Cardiff CF14 4XN, UK; 5 Section of Pathology, Institute of Cancer and Genetics, Cardiff University, School of Medicine, Heath Park, Cardiff CF14 4XN, UK

## Abstract

Fibrosis in response to tissue damage or persistent inflammation is a pathological hallmark of many chronic degenerative diseases. By using a model of acute peritoneal inflammation, we have examined how repeated inflammatory activation promotes fibrotic tissue injury. In this context, fibrosis was strictly dependent on interleukin-6 (IL-6). Repeat inflammation induced IL-6-mediated T helper 1 (Th1) cell effector commitment and the emergence of STAT1 (signal transducer and activator of transcription-1) activity within the peritoneal membrane. Fibrosis was not observed in mice lacking interferon-γ (IFN-γ), STAT1, or RAG-1. Here, IFN-γ and STAT1 signaling disrupted the turnover of extracellular matrix by metalloproteases. Whereas IL-6-deficient mice resisted fibrosis, transfer of polarized Th1 cells or inhibition of MMP activity reversed this outcome. Thus, IL-6 causes compromised tissue repair by shifting acute inflammation into a more chronic profibrotic state through induction of Th1 cell responses as a consequence of recurrent inflammation.

## Highlights

• Repeated acute resolving inflammation leads to excessive tissue damage
• IL-6 regulates profibrotic IFN-γ-secreting T cells
• IFN-γ increases detrimental STAT1 signaling in stromal tissue
• STAT1 activity alters homeostatic control of extracellular matrix to promote fibrosis

## Introduction

Fibrosis of connective tissues or organ structures is characterized by alterations in extracellular matrix deposition as a consequence of tissue damage or persistent inflammation (Wynn, 2007). Acquired loss of peritoneal function as a result of fibrosis is a major factor leading to ultrafiltration and treatment failure in renal patients on peritoneal dialysis. Thickening of the submesothelial compact zone is commonly linked with both treatment duration and the incidence of bacterial peritonitis in this patient group (Davies et al., 1996, Davies et al., 2001, Williams et al., 2002). Here, increased peritoneal fibrosis corresponds with the severity of infection and the number of episodes encountered (Davies et al., 1996). The cellular mechanisms initiating this response are currently unclear.

During acute infection, leukocyte infiltration is tightly regulated to ensure both bacterial clearance and the successful resolution of inflammation (Jones, 2005). In contrast, localized persistent or recurrent infections promote tissue injury and fibrogenesis (Casadevall and Pirofski, 2003). Here, fibrosis is associated with retention of an activated leukocyte population within the infected organ. Inflammatory cytokines including interleukin-4 (IL-4), IL-13, transforming growth factor-β (TGF-β), and oncostatin-M have all been linked to the development of fibrosis in autoimmune conditions such as systemic sclerosis or interstitial lung disease (Mozaffarian et al., 2008, Roberts et al., 1986, Sempowski et al., 1994, Zhu et al., 1999). However, their roles and the roles of other cytokines in peritoneal fibrosis have not yet been examined.

Interleukin-6, acting via the latent transcription factors signal transducer and activator of transcription-3 (STAT3) and STAT1, plays pivotal roles in governing leukocyte infiltration during acute inflammation (Fielding et al., 2008, Hurst et al., 2001, Jones et al., 2011, McLoughlin et al., 2003, McLoughlin et al., 2005). These findings may relate to the involvement of IL-6 in antimicrobial host defense and the inability of *Il6*^−/−^ mice to effectively clear bacterial or viral infections (Detournay et al., 2005, Dienz et al., 2012, Doganci et al., 2005, Dominitzki et al., 2007, Finotto et al., 2007, Kopf et al., 1994, Lee et al., 1999, Longhi et al., 2008, McLoughlin et al., 2005, Murphy et al., 2008, Pasare and Medzhitov, 2003, Quinton et al., 2008, Yamamoto et al., 2000). In contrast, inflammatory models of chronic disease and clinical observations identify IL-6 activity as deterimental in autoimmunity and cancer (Jones, 2005, Jones et al., 2011, Nishimoto and Kishimoto, 2004). It is, however, unknown how IL-6 converts from providing protective immunity to its damaging activities that drive chronic inflammation and fibrosis.

We have previously described a peritoneal model of inflammation based on the administration of a cell-free supernatant prepared from a clinical isolate of *Staphylococcus epidermidis* (termed SES). This model closely mimicks a resolving inflammatory response typically seen in clinical bacterial peritonitis (Hurst et al., 2001, McLoughlin et al., 2003). Through adaptation of this model, we now show that repeated SES challenge promotes peritoneal fibrosis in wild-type (WT) mice. This response strictly required IL-6, which regulated a T-cell-mediated increase in tissue damage and membrane fibrosis. These data suggest that IL-6 blocking interventions may be useful in the treatment of infection-associated fibrotic conditions and support the potential prognostic value of monitoring IL-6-directed “STAT1 signatures” in dialysis patients.

## Results

### Peritoneal Fibrosis after Recurrent SES Inflammation Requires IL-6 Signaling

**Figure 1:**
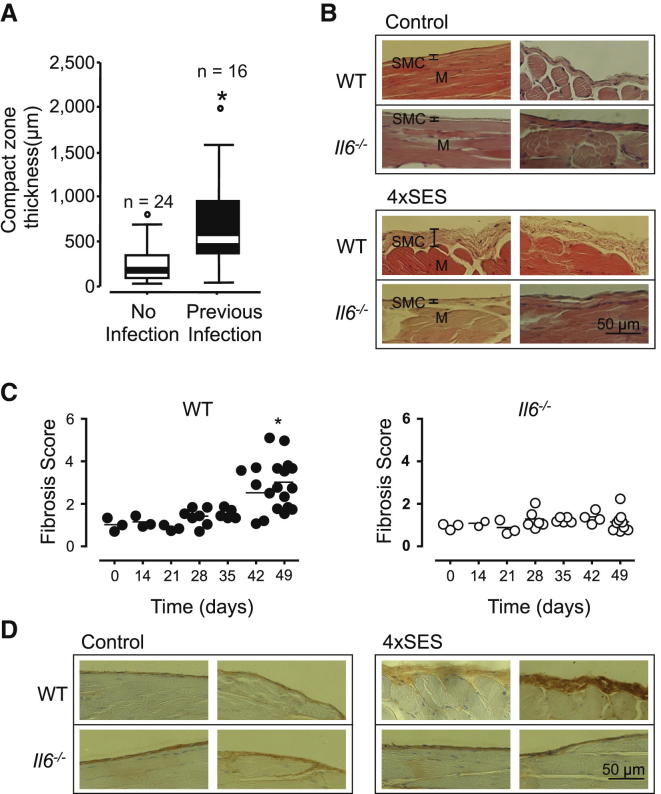
*Il6*^−/−^ Mice Are Protected from the Development of Fibrosis after Repeated Inflammation (A) Submesothelial compact zone thickness was compared in peritoneal biopsies from PD patients with either no previous infection history or a defined infection history. Data are presented as box plots of the interquartile range (IQR). Lines extend from the box to the highest and lowest values, excluding outliers. The median value is represented by a thick line across each box. (B) WT or *Il6*^−/−^ mice were injected (i.p.) with SES at weekly intervals for 3 weeks (day 0–21) and left for a further 4 weeks until day 49 before histological analysis of the peritoneal membrane. Peritoneal membrane sections (5 μm) taken from SES-treated and age-matched control mice on day 49 were stained with hematoxylin and eosin and examined for submesothelial compact zone thickening (layer between the muscle and membrane surface). Representative fields are shown from two individual mice per group (×400 magnification). Scale bar represents 50 μM. Submesothelial compact zone (SMC) and muscle layers (M) are indicated on representative WT sections. (C) Fibrosis scores for WT and *Il6*^−/−^ mice are shown over the duration of the model. Values reflect the fold-change in submesothelial zone thickness compared to the WT control group at day 0 (n ≥ 3–12 per group, unpaired t test ^∗^p < 0.05 compared to WT day 0, ^∗∗^p < 0.001 compared to WT control day 49 and *Il6*^−/−^ 4×SES day 49). No significant difference in membrane thickening was observed in sections from WT control mice taken on day 0 and day 49. (D) Peritoneal membrane sections from day 49 were immunostained with antibodies against type-1 collagen. Representative fields are shown from two individual mice per group (×400 magnification). Scale bar represents 50 μm. See also Figure S1.

Prolonged peritoneal dialysis (PD) treatment leads to alterations in peritoneal membrane function and tissue fibrosis (Williams et al., 2002). Histological assessment of biopsies taken from the peritoneal membrane of PD patients show that those who have never experienced a peritonitis episode display less thickening of the submesothelial compact zone than those that had encountered at least one prior infection (Figure 1A). To evaluate the relationship between infection incidence and peritoneal fibrosis, a model of recurrent peritoneal inflammation was developed through administration of a cell-free supernatant prepared from of a clinical isolate of *Staphylococcus epidermidis* (termed SES) (Figure S1 available online). Mice were challenged (i.p.) at 7 day intervals with four sequential rounds of acute SES-induced inflammation. Parietal peritoneal membrane sections were prepared at various time points after resolution of the fourth inflammatory episode (Figures 1B, 1C, and S1). Consistent with the histology of human parietal peritoneum, unchallenged WT mice showed two distinct cellular regions: an underlying area of muscle and a mesothelial monolayer on the surface of a thin basal lamina (a submesothelial compact zone) (Williams et al., 2002).

Repeat SES activation in WT mice caused a distinct thickening of the submesothelial compact zone, which emerged at day 35 after four rounds of inflammation (Figures 1B and 1C). This was further illustrated by immunohistochemistry of collagen type-1 (Figures 1D and S1). Previous investigations with a single administration of SES showed that IL-6 controls leukocyte recruitment and influences the course of acute resolving peritoneal inflammation (Fielding et al., 2008, Hurst et al., 2001, Jones et al., 2011, McLoughlin et al., 2003, McLoughlin et al., 2005). We therefore monitored the onset of fibrosis in SES-challenged *Il6*^−/−^ mice. Peritoneal membranes from *Il6*^−/−^ mice showed no thickening of the submesothelial compact zone after repeat SES activation. There was also no alteration in type-1 collagen deposition and sections closely resembled those taken from nonchallenged mice (Figures 1B–1D and S1).

To test whether the degree of pathology observed was due to the intensity or duration of inflammation, we evaluated fibrosis in SES-challenged *Il10*^*−/−*^ mice. Here, IL-10 deficiency caused a substantial increase in leukocyte infiltration, cytokine production (including IL-6), and T cell effector function after SES activation (Figure S2). Despite this increase in inflammatory activation, *Il10*^*−/−*^ mice showed a similar degree of fibrosis to that of WT mice (Figure S2). Thus, the severity of peritoneal fibrosis is independent of the intensity of acute inflammatory activation.

### Peritoneal Fibrosis Was Not Attributable to Increases in Profibrotic Cytokines or IL-6

To determine whether fibrosis in WT mice was attributable to an increase in IL-6 bioavailability as a response to repeat inflammatory activation, IL-6 and its soluble receptor (sIL-6R) were quantified in peritoneal lavage from the first and fourth episode of SES-induced inflammation (Figure S3). No difference in IL-6 or sIL-6R expression was observed as a consequence of repeated inflammation. Next, we quantified changes in several profibrotic cytokines. IL-4 remained below the limit of detection, and only small SES-induced changes in IL-13 were observed (data not shown). A similar pattern of IL-4 and IL-13 production was also observed in vitro (Figure S3). Here, quantification of IL-4 and IL-13 in conditioned supernatants from SES-challenged peritoneal monocytic cells showed no IL-4, but some expression of IL-13, that was not enhanced by the addition of IL-6. Finally, we considered the potential role of TGF-β, which is a major profibrotic cytokine that signals via the transcription factor SMAD3 (Wynn, 2008). Although small differences in TGF-β expression was observed between SES-challenged WT and *Il6*^−/−^ mice, TGF-β amounts remained largely unaltered by repeated SES challenge (Figure S3). Importantly, *Smad3*^*−/−*^ mice were not protected from SES-induced peritoneal fibrosis (Figure S3), suggesting that TGF-β signaling is not required for the observed fibrotic changes. Thus, cytokines traditionally viewed as profibrotic are not integral to the development of SES-induced peritoneal fibrosis.

### Recurrent Inflammation Drives Increased STAT1 Signaling in the Peritoneal Membrane

**Figure 2:**
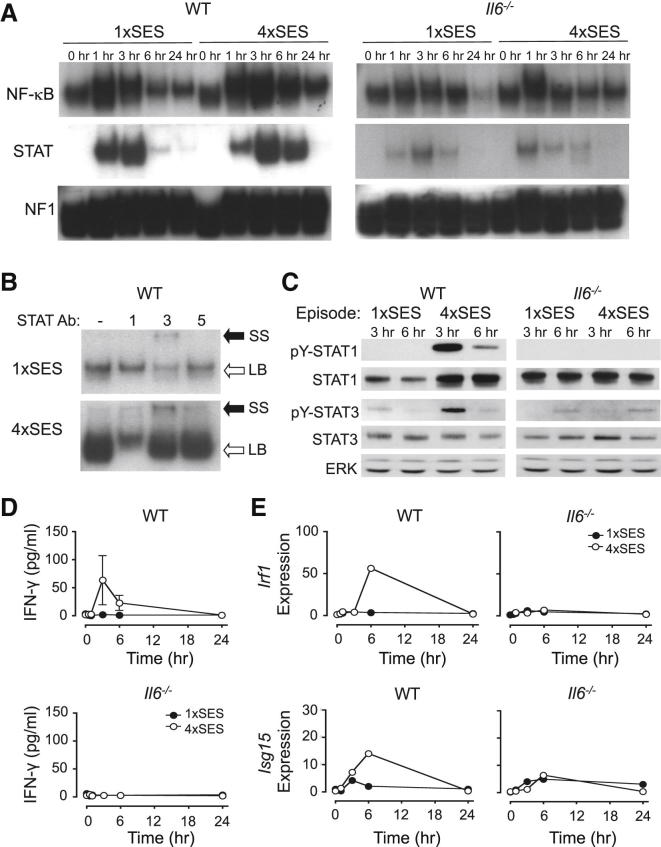
Changes in the Inflammatory Response after Repeated SES Challenge (A) EMSA for NF-κB and STAT signaling in nuclear extracts from the peritoneum of SES-challenged WT and *Il6*^−/−^ mice. Analysis of samples from the first (1×SES) and fourth (4×SES) episode of inflammation are shown. An NF1 probe was used as a loading control. (B) Supershift analysis of the STAT DNA-binding complex in nuclear extracts (3 hr sample) from WT mice. Analysis of STAT1, STAT3, and STAT5 is shown from 1×SES and 4×SES. The antibody-induced STAT3 supershift (SS) and loss of STAT1 binding to the probe (LB) are indicated by black and white arrows, respectively. (C) Immunoblot with phospho-specific antibodies for STAT1 and STAT3 in protein lysates from the peritoneal membranes of mice. Samples were obtained from 1×SES and 4×SES. In all cases, results are representative of lysates from three different mice per time point and genotype. (D) Detection of IFN-γ in lavage from challenged WT and *Il6*^−/−^ mice (mean ± SEM; n > 5 per time point). (E) qPCR of *Irf1* and *Isg15* in total RNA from the peritoneal membrane of challenged mice. Values are expressed relative to the WT baseline control and represent the mean for each genotype from three different mice per time. See also Figure S4.

SES is a potent activator of TLR2, which signals through NF-κB (Colmont et al., 2011). We therefore compared SES activation of NF-κB in WT and *Il6*^−/−^ mice via electrophoretic mobility shift assays (EMSA) (Figure 2A). Analysis of nuclear extracts from the peritoneal membrane revealed that a single dose of SES triggered a temporal activation of NF-κB, which was sustained over a 12–24 hr period. Minimal NF-κB activity was observed at baseline (time zero for either episode 1 or 4), suggesting that the inflammatory signal was resolved after each round of SES activation (Figure 2A). Although NF-κB activity was enhanced in mice encountering their fourth inflammatory episode, there was no appreciable difference between WT and *Il6*^−/−^ mice (Figures 2A and S4). Supershift analysis of the DNA-protein complex confirmed that NF-κB was unaffected by IL-6 deficiency and uniformly consisted of both p65 (Rel-A) and p50 subunits (Figure S4). Thus, WT and *Il6*^−/−^ mice display similar SES responsiveness, which suggests that the signals driving fibrosis are downstream of TLR2-mediated NF-κB activation.

We next considered IL-6 activation of STAT1 and STAT3 by EMSA. Whereas nuclear extracts from SES-challenged WT mice showed a robust STAT response, this activity was markedly impaired in *Il6*^−/−^ mice (Figures 2A, 2B, and S4). In WT mice, STAT activity was transient and began to resolve at 6 hr after SES administration. STAT activation coincided with the detection of *Il6* in total mRNA from peritoneal extracts of SES-challenged WT mice (Figure S3). The intensity and duration of STAT signaling was, however, substantially increased in the fourth SES-driven episode (Figure 2A). Supershift analysis, via a STAT1 antibody (which causes a loss of DNA binding [LB]) and a STAT3 antibody (which induces a classical supershift [SS] in electrophoretic mobility), showed that the composition of the DNA-STAT protein complex was altered as a consequence of recurrent SES challenge (Figures 2B and S4). In the first acute SES challenge, the DNA-protein complex was predominantly composed of STAT3, with little evidence of STAT1. In contrast, there was a notable shift toward STAT1 over STAT3 activation in nuclear extracts taken during the fourth SES episode (Figure 2B). This increase in STAT1 activity was confirmed by immunoblot of peritoneal extracts via antibodies against tyrosine-phosphorylated STAT1 and STAT3 (Figure 2C). Thus, prior inflammatory activation causes an alteration in STAT signaling capacity and leads to an increase in STAT1 activity.

### Increased IFN-γ Promotes STAT1 Activation and Associated Target Gene Expression

The absence of STAT activity in peritoneal extracts from SES-challenged *Il6*^−/−^ mice (Figure 2) infers that the increase in STAT1 signaling seen in WT tissue could arise from either altered IL-6 and gp130-mediated signaling or through IL-6 regulation of a downstream STAT1 activating factor. Because prior experiments with SES administration supported an inflammatory interplay between IL-6 and IFN-γ (McLoughlin et al., 2003), IFN-γ was quantified in the first and fourth round of SES challenge (Figure 2D). No IFN-γ was detected in the peritoneal cavity of WT or *Il6*^−/−^ mice after a single SES dose (Figure 2D). In contrast, IFN-γ was markedly elevated in WT mice during the fourth SES challenge. These changes coincided with increases in STAT1 activity and the induction of STAT1 target genes (*Irf1*, interferon regulatory factor-1; *Isg15*, ubiquitin-like modifier interferon stimulated gene-15) (Figures 2B, 2C, and 2E). No change in IFN-γ expression and STAT1 signaling was observed in *Il6*^−/−^ mice (Figures 2D and 2E). Thus, IL-6 signaling may promote an increase in IFN-γ activity after repeated inflammatory challenge.

### Impaired IFN-γ and STAT1 Signaling Prevents Peritoneal Fibrosis

**Figure 3:**
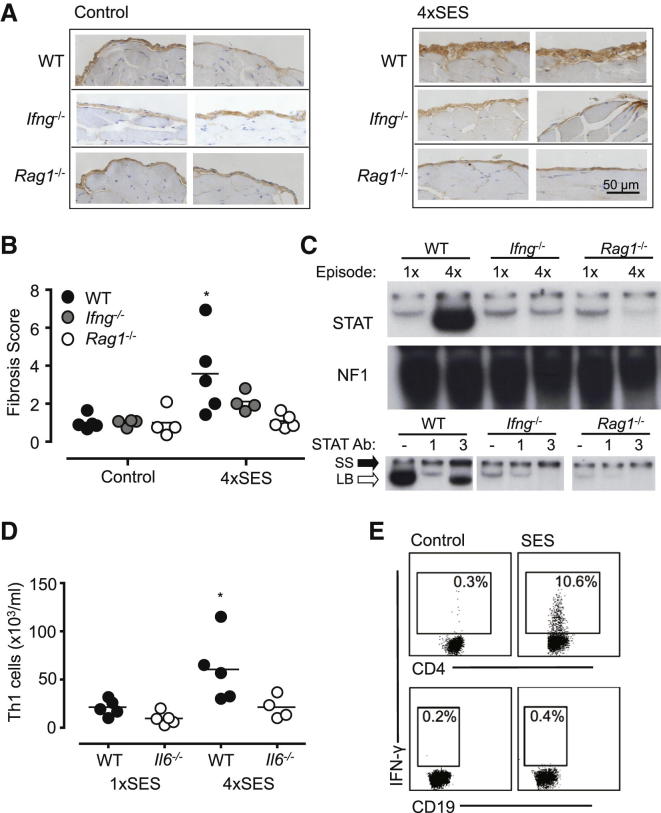
Detection of Peritoneal IFN-γ-Expressing CD4^+^ T Cells Correspond with STAT1 Activation (A) WT, *Ifng*^*−/−*^, and *Rag1*^*−/−*^ mice were repeatedly challenged with SES and the peritoneal membrane harvested at day 49. Serial sections were immunostained for type-1 collagen and counterstained with hematoxylin. Representative fields are shown from two individual mice per group (×400 magnification). (B) Fibrosis was scored by assessment of the fold-change in the submesothelial compact zone thickness compared to the control group at day 49 (n ≥ 4 per group unpaired t test; ^∗^p < 0.05). (C) STAT activation at 3 hr post-SES injection was measured by EMSA of nuclear extracts prepared from the peritoneal membrane of WT, *Ifng*^*−/−*^, and *Rag1*^*−/−*^ mice taken during 1×SES and 4×SES. Supershift analysis of nuclear extracts from 1×SES and 4×SES by specific anti-STAT antibodies. Data are representative of results from three mice. The antibody-induced STAT3 supershift (SS) and loss of STAT1 binding to the probe (LB) are indicated by black and white arrows, respectively. (D) Intracellular flow cytometry for IFN-γ-secreting CD4^+^ T cells in peritoneal lavage from SES-challenged WT and *Il6*^−/−^ mice. Lavage samples were isolated at 3 hr during 1×SES and 4×SES (n ≥ 4 per group, unpaired t test; ^∗^p < 0.05 compared with the 1×SES WT group). (E) Peritoneal leukocytes obtained from WT mice (6 hr after SES) were stimulated ex vivo for a further 4 hr with SES in the presence of monensin. Intracellular flow cytometry for IFN-γ production in CD4^+^ and CD19^+^ lymphocytes is shown. See also Figure S5.

To define a link between changes in IFN-γ and peritoneal fibrosis, *Ifng*^*−/−*^ mice were repeatedly challenged with SES (Figure 3A). Peritoneal histology from *Ifng*^*−/−*^ mice showed limited collagen type-1 staining and were protected from fibrotic thickening (Figures 3A and S5).

Because lymphoid cells are a major source of IFN-γ, we next evaluated SES-induced fibrosis in *Rag1*^*−/−*^ mice. No peritoneal fibrosis was seen in RAG-1 deficiency (Figures 3A and 3B). To examine the potential relationship between IFN-γ-secreting lymphoid cells and the transition toward a STAT1-mediated response, STAT signaling was examined in both *Ifng*^*−/−*^ and *Rag1*^*−/−*^ mice after SES administration. No increase in stromal STAT1 signaling was observed in the peritoneum of SES-challenged *Ifng*^*−/−*^ and *Rag1*^*−/−*^ mice (Figures 3C and S5). Importantly, although SES-induced changes in peritoneal IL-6 were comparable in WT, *Ifng*^*−/−*^, and *Rag1*^*−/−*^ mice, IFN-γ remained below detection in *Ifng*^*−/−*^ and *Rag1*^*−/−*^ mice (Table 1). Consequently, IL-6 does not directly promote peritoneal fibrosis. Instead, IL-6 control of IFN-γ may account for the changes in tissue damage.

**Table 1:** IL-6 and IFN-γ Production in WT, *Ifng*^*−/−*^, and *Rag1*^−/−^ Mice

	**Episode 1**				**Episode 4**			
	**0 hr**		**3 hr**		**0 hr**		**3 hr**	
	**IL-6**	**IFN-γ**	**IL-6**	**IFN-γ**	**IL-6**	**IFN-γ**	**IL-6**	**IFN-γ**
**WT**	5 ± 3	<LD	590 ± 199^∗^	<LD	2 ± 2	<LD	284 ± 32^∗∗^	24 ± 4
***Ifng***^***−/−***^	16 ± 11	<LD	451 ± 205	<LD	<LD	<LD	152 ± 39^∗^	<LD
***Rag1***^**−/−**^	29 ± 7	<LD	1,057 ± 514^∗^	<LD	<LD	<LD	938 ± 352	<LD

IL-6 and IFN-γ protein production (pg/ml) was measured in WT, *Ifng*^*−/−*^, and *Rag1*^−/−^ mice by specific ELISA within peritoneal lavage fluid. ELISA results shown are the mean ± SEM (n ≥ 4 per time point). <LD indicates below the limit of detection. ^∗^p < 0.05 IL-6 levels WT episode 1, 0 hr versus 3 hr; *Rag1*^−/−^ episode 1, 0 hr versus 3 hr; WT versus *Ifng*^−/−^ and *Ifng*^−/−^ versus *Rag1*^−/−^ episode 4, 3 hr; and ^∗∗^p < 0.0001 WT episode 4, 0 hr versus 3 hr.

### Increases in IFN-γ-Secreting T Cells Control STAT1 Signaling in the Peritoneal Membrane

Our data show that IL-6 promotes an increase in IFN-γ and STAT1 signaling as a consequence of repeat SES challenge. This response is associated with increased numbers of lymphocytes and monocytes in peritoneal lavage (Table 2). Because IFN-γ expression is restricted to lymphoid cells, we quantified IFN-γ-secreting CD4^+^ T cells (Th1 cells) in WT and *Il6*^−/−^ mice. In WT mice after the fourth SES administration (3 hr after SES challenge), there was a notable increase in overall Th1 cell numbers. No increase in Th1 cells was seen in *Il6*^−/−^ mice (Figure 3D). Peritoneal IL-17-secreting CD4^+^ T cells (Th17 cells) were always 10-fold lower than Th1 cells in WT mice, and very few IL-17, IFN-γ double-positive T cells were detected (Jones et al., 2010). Examination of effector CD4^+^ T cells during the period associated with the development of peritoneal fibrosis (days 28–49) showed that this Th1 cell phenotype was maintained and represented the most prominent CD4^+^ T cell population within the peritoneal cavity (Figure S5).

**Table 2:** Resident Peritoneal Leukocyte Counts after Repeated Inflammation

Leukocytes (×10^6^/ml)	WT		*Il6*^−/−^	
	Episode 1	Episode 4	Episode 1	Episode 4
Macrophages	2.42 ± 0.18	4.10 ± 0.54^∗^	2.35 ± 0.22	4.07 ± 0.48^∗^
PMNs	0 ± 0	0.27 ± 0.25	0 ± 0	0.07 ± 0.04
Lymphocytes	0.20 ± 0.06	1.59 ± 0.32^∗∗^	0.59 ± 0.06	1.37 ± 0.07^∗∗^

Resident populations of peritoneal leukocytes were analyzed by differential cell counting during the first and fourth episodes of SES-induced peritoneal inflammation in WT and *Il6*^−/−^ mice (mean ± SEM; n ≥ 5 per group, unpaired t test, ^∗^p < 0.05 or ^∗∗^p < 0.01).

Recent studies suggest that innate B cell activation causes the release of certain inflammatory cytokines (Barr et al., 2007, Barr et al., 2010). CD19^+^ B cells from SES-challenged mice did not produce IFN-γ after restimulation in vitro with SES (Figure 3E). These studies preclude a role for innate IFN-γ production by B cells. However, they do not eliminate the involvement of IL-6-driven B cell antibody generation (Hirano et al., 1986), which in turn may drive antibody-mediated tissue injury. Consequently, an ELISA was developed to evaluate the generation of anti-SES IgG (Figure S4). Repeated SES-induced inflammation caused significant increases in anti-SES IgG titers. The antibodies were highly polyclonal and immunoblot analysis showed them to bind multiple components within the SES lysate (Figure S6i). Similar increases in anti-SES IgG were detected in both *Il6*^−/−^ and *Ifng*^*−/−*^ mice (Figure S6i). Thus, fibrosis is not driven by alterations in B cell antibody generation.

### SES Triggers the IL-6-Dependent Expansion of Th1 Cells In Vitro

**Figure 4:**
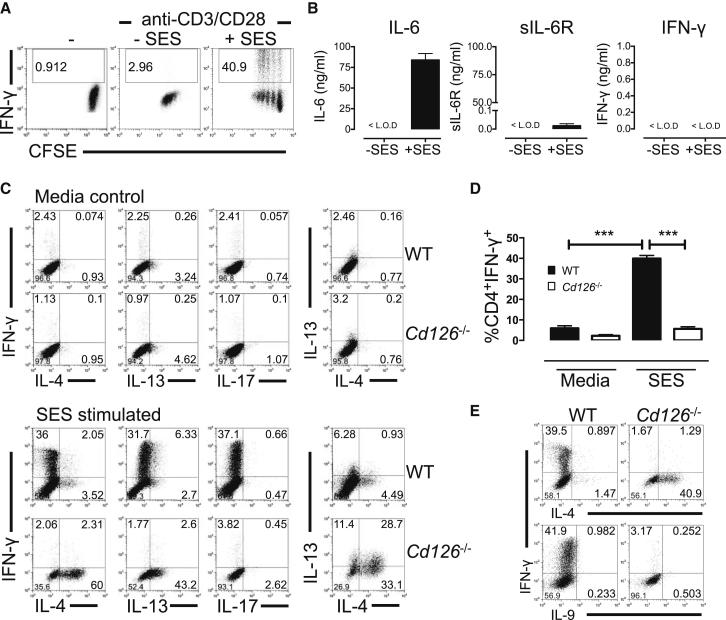
SES Promotes the IL-6-Dependent Expansion of Th1 Cells In Vitro (A) Peritoneal monocytic cells were recovered by lavage from WT mice and stimulated with SES in culture overnight. Cell-free conditioned media from these cultures (SES-CM) were used to stimulated CFSE-labeled naive T cells under anti-CD3 and anti-CD28 costimulation. After 4 days culture, IFN-γ production was monitored in proliferating CD4^+^ T cells. Representative data are shown for cells treated with media alone (-), costimulatory antibodies alone (-SES), or in combination with SES-CM (+SES). (B) ELISA quantification of IL-6, sIL-6R, and IFN-γ in SES-CM (mean ± SEM; n = 3 for IL-6 and IFN-γ, n = 6 for sIL-6R; values below the limit of detection [<L.O.D.] are indicated). (C) Intracellular flow cytometry for cytokine production by naive CD4^+^ T cells cultured for 4 days under anti-CD3 and anti-CD28 costimulation in the presence or absence of SES-CM. Data are shown for T cells derived from WT and *Cd126*^*−/−*^ mice. (D) Relative quantification of IFN-γ-secreting CD4^+^ T cells in all experimental repeats (n = 5 WT and n = 3 *Cd126*^*−/−*^ mice per group). (E) Comparable analysis of IFN-γ, IL-4, and IL-9 in T cell cultures from WT and *Cd126*^*−/−*^ mice. See also Figure S6i.

To evaluate the mechanism of Th1 cell expansion, peritoneal monocytic cells were isolated from WT mice and stimulated in vitro with SES. Conditioned medium from these cultures (SES-CM) was then added to naive CD4^+^ T cells activated by anti-CD3 and anti-CD28 costimulation. Addition of SES-CM caused a proliferative expansion of IFN-γ-secreting CD4^+^ T cells (Figure 4A). Whereas large quantities of IL-6 were detected in SES-CM, sIL-6R was barely detectable (<20 pg/ml after SES activation) and IFN-γ remained below the limit of detection (Figure 4B). To test whether IL-6 was responsible for driving the Th1 cell response, naive CD4^+^ T cells from WT and *Cd126*^*−/−*^ mice (deficient in IL-6Rα) were cultured with costimulatory antibodies and SES-CM. WT CD4^+^ T cells displayed a robust Th1 cell signature (Figures 4C and 4D) and did not produce IL-4, IL-9, IL-13, or IL-17 (Figures 4C and 4E). In contrast, SES-CM caused no increase in IFN-γ production in *Cd126*^*−/−*^ T cells. Instead, the absence of IL-6Rα enhanced development of IL-4- and IL-13-producing T cells, with no change in IL-9 or IL-17 (Figures 4C and 4E). These data suggest that IL-6 acting directly via IL-6Rα controls the differentiation and expansion of Th1 cells.

**Figure 5:**
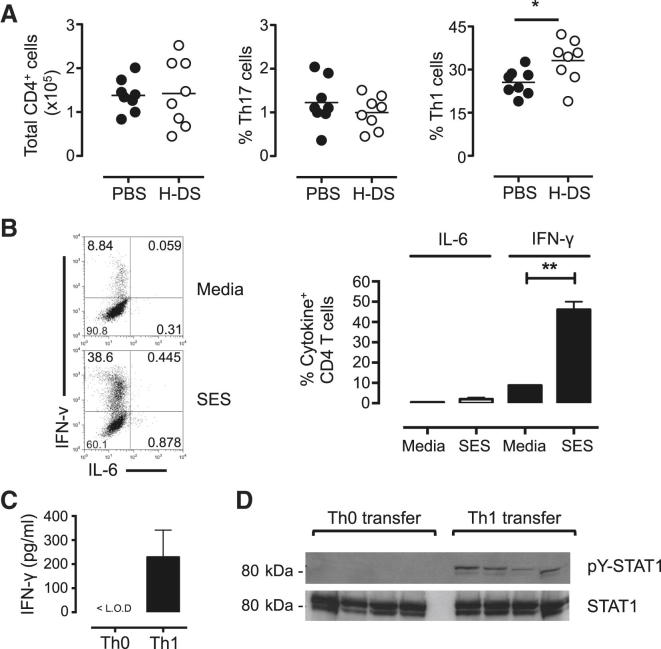
IL-6 Modulation of Th1 Cell Activity In Vivo (A) SES-induced peritoneal inflammation was initiated in *Il6*^−/−^ mice. Local IL-6 (*trans*) signaling was reconstituted via administration (i.p.) of 1 μg/mouse HDS or control PBS at 0 hr (same time as SES challenge), 24 hr, and 48 hr. After 72 hr, the peritoneal infiltrate was recovered by lavage and CD4^+^ T cells examined by flow cytometry. The proportion of CD4^+^ T cells displaying a Th1 and Th17 effector cell phenotype was determined by intracellular flow cytometry for IFN-γ and IL-17A (n = 8 mice per treatment, p < 0.05). (B) Intracellular flow cytometry of IFN-γ and IL-6 production by naive CD4^+^ T cells from WT mice that had been activated for 4 days in the presence or absence of SES-CM from peritoneal monocytic cells. The relative quantification of cells releasing IL-6 or IFN-γ is presented from all experiments (n = 3, p < 0.05). (C) WT Th1 cells expanded ex vivo under costimulation with SES-CM. These Th1 cells (0.5–1.0 × 10^6^) were adoptively transferred (i.p.) into *Cd126*^*−/−*^ mice together with SES. Peritoneal lavage were recovered (3 hr) and IFN-γ quantified by ELISA (mean ± SEM from four separate mice). Values are compared against mice receiving freshly sorted naive CD4^+^ T cells (Th0). (D) Immunoblot of STAT1 activation in peritoneal membranes from SES-challenged *Cd126*^*−/−*^ mice receiving ex vivo expanded Th1 cells or Th0 cells. Data are shown for each individual adoptive transfer (n = 4). See also Figure S6ii.

To determine how IL-6Rα signaling may influence Th1 cell development, we next evaluated the impact of IL-6 on IL-12, a recognized activator of Th1 cell commitment. Although IL-12 induction by SES was comparable in peritoneal monocytic and dendritic cells from WT and *Il6*^−/−^ mice, the presence of IL-6 may influence the commitment of Th1 cells by IL-12 (Figures S3 and S7). Consistent with previous studies (Diehl et al., 2000, Rincón et al., 1997), IL-6 suppressed IL-12 regulation of Th1 cell differentation (Figure S6ii). However, IL-6 was essential for T cell survival and this led to an overall increase in Th1 cell numbers in IL-12-treated cultures (Figure S6ii). To test whether IL-6 could influence the control of T cell effector function in vivo, IL-6 signaling was reconstituted in SES-challenged *Il6*^−/−^ mice through administration (i.p.) of an IL-6/sIL-6R fusion protein (HDS). Here, HDS treatment led to a significant increase in the frequency of Th1 cells at 72 hr after SES administration (Figure 5A). Thus, IL-6 promotes T cell survival to maintain their effector characteristics within the peritoneal cavity.

### In Vitro Committed Th1 Cells Promote Stromal STAT1 Activation and Peritoneal Fibrosis In Vivo

To identify a link between T cell-derived IFN-γ and peritoneal fibrosis, WT T cells expanded in vitro with SES-CM were transferred into *Cd126*^*−/−*^ mice. With *Cd126*^*−/−*^ mice as recipients, the only cell type capable of responding to IL-6 is the transferred T cell population. Although in vitro expanded CD4^+^ T cells secreted IFN-γ, IL-6 was not detected in these cultures (Figure 5B). When these Th1 cells were transferred (i.p.) into *Cd126*^*−/−*^ mice, increases in both peritoneal IFN-γ and stromal STAT1 activity were detected. Transfer of control naive (Th0) CD4^+^ T cells had no affect (Figures 5C and 5D).

**Figure 6:**
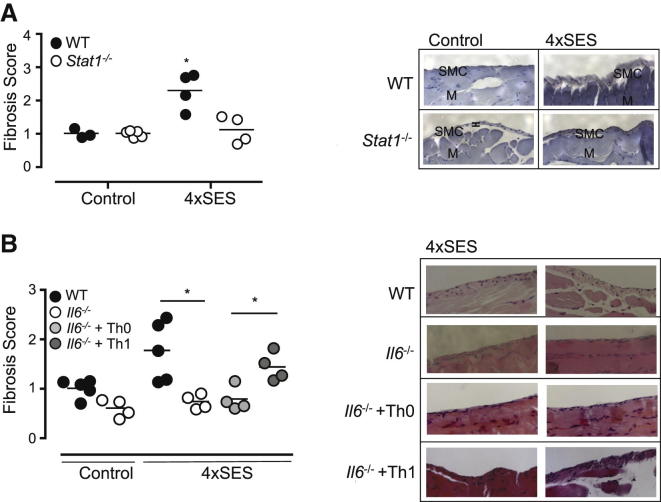
Peritoneal Fibrosis Is Linked to Stromal STAT1 Activity and Th1 Cells (A) WT and *Stat1*^*−/−*^ mice were repeatedly challenged with SES. Peritoneal membranes were harvested at day 49 and sections scored for fibrosis. Sections are compared against age-matched control mice as before (unpaired t test, ^∗^p < 0.05). (B) Naive CD4^+^ T cells were committed in vitro to Th1 cells with SES-CM. The proportion of IFN-γ^+^CD4^+^ cells was determined by flow cytometry and used to calculate the number of T cells for transfer into *Il6*^−/−^ mice. 0.5–1.0 × 10^6^ Th1 cells or naive CD4^+^ T cells (Th0) were coadministered to mice with SES (i.p.) on day 0, 7, 14, and 21. Peritoneal fibrosis was assessed on day 49 as before (unpaired t test, ^∗^p < 0.05). See also Figure S7i.

Studies next tested the importance of STAT1 activation as a prerequisite to the development of peritoneal fibrosis. When compared to WT mice, *Stat1*^*−/−*^ mice showed no signs of fibrosis after repeat SES activation (Figures 6A and S7i). To demonstrate a role for Th1 cells in the control of peritoneal fibrosis, Th1 cells expanded with SES-CM were adoptively transferred into the peritoneal cavity of *Il6*^−/−^ mice. Freshly expanded Th1 cells (0.5–1.0 × 10^6^ cells, corrected for the proportion secreting IFN-γ) or an equivalent number of naive (Th0) CD4^+^ T cells were administered together with each dose of SES (Figure S7i). Transfer of Th1 cells, but not naive CD4^+^ T cells, induced peritoneal fibrosis in *Il6*^−/−^ mice (Figure 6B).

### IFN-γ and STAT1 Regulate Matrix Metalloproteinase Expression In Vitro and In Vivo

**Figure 7:**
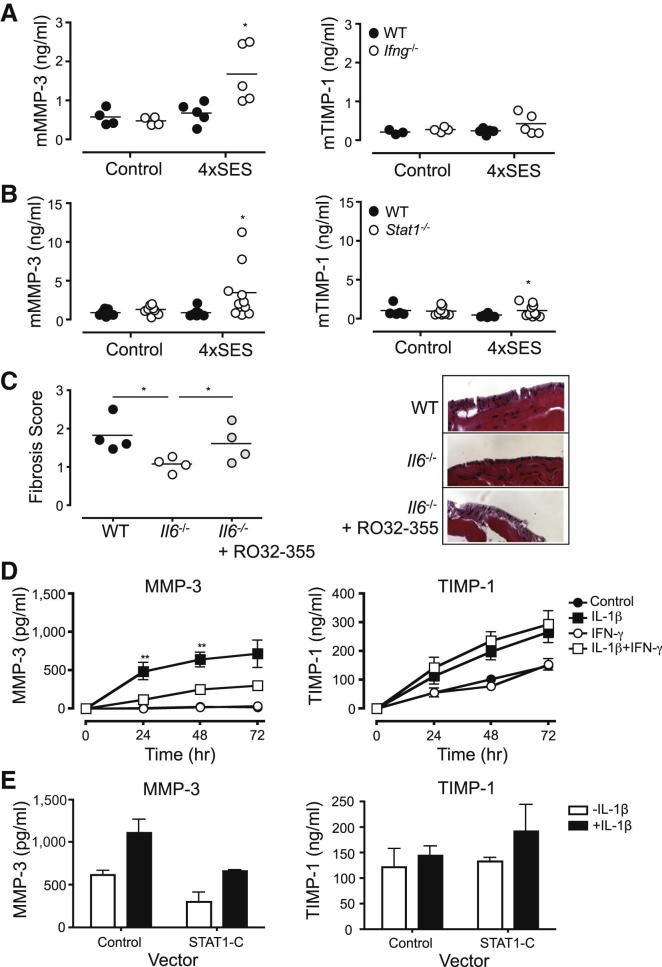
IFN-γ-STAT1 Activity Regulates Matrix Metalloproteinase Expression (A and B) ELISA quantification of MMP-3 and TIMP-1 in peritoneal lavage fluid from WT and *Ifng*^*−/−*^ (A) or *Stat1*^*−/−*^ (B) mice. (C) WT and *Il6*^−/−^ were injected with SES at four weekly intervals. At day 43, day 45, and day 47, *Il6*^−/−^ mice received drinking water containing the collagenase-specific MMP inhibitor Ro32-355 (12.5 mg/50 ml). Groups of WT and *Il6*^−/−^ mice received the ethanol vehicle alone. At day 49, the peritoneal membrane was harvested and fibrosis scored as before. (D) Growth-arrested human peritoneal mesothelial cells (HPMCs) were treated with medium alone (control), IL-1β (100 pg/ml), IFN-γ (100 U/ml), or IL-1β in combination with IFN-γ for up to 72 hr. Cell-free supernatants were analyzed for MMP-3 or TIMP-1 by ELISA. (E) HPMCs were transfected with empty control or constitutive STAT1 (STAT1-C) containing plasmid vectors overnight and stimulated with IL-1β (100 pg/ml) for 24 hr. Cell-free supernatants were analyzed for MMP-3 or TIMP-1 by ELISA. See also Figure S7ii.

Fibrosis is typically associated with altered patterns of matrix degradation, where increased stromal cell proliferation leads to deposition of extracellular matrix proteins and scarring (Wynn, 2007). The regulation of matrix metalloproteases (MMP) or tissue inhibitors of MMP (TIMP) by IFN-γ might therefore provide a mechanism for the observed peritoneal fibrosis. To test this, peritoneal changes in MMP-3 was examined in WT versus *Ifng*^*−/−*^ and *Stat1*^*−/−*^ mice (Figures 7A and 7B). Here, IFN-γ or STAT1 deficiency led to an increase in MMP-3 expression in mice repeatedly challenged with SES. No alteration in peritoneal TIMP-1 was observed. Thus, enhanced IFN-γ and STAT1 signaling might limit MMP activity to promote the deposition of extracellular matrix. To demonstrate a link between MMP activity and the protection from fibrosis observed in *Il6*^−/−^ mice, a collagenase-specific MMP inhibitor (Ro32-355) was administered (between days 43 and 49) orally to *Il6*^−/−^ mice after the fourth round of SES-induced inflammation (Figure 7C). Under these conditions, *Il6*^−/−^ mice developed comparable fibrosis to that seen in WT mice.

To see whether a similar mechanism may account for fibrosis in PD patients, a series of in vitro studies were performed. First, primary human peritoneal mesothelial cells (HPMCs) were stimulated with IL-1β (a major regulator of MMP production) and secretion of MMP-3 and TIMP-1 was assessed in the presence of IFN-γ. As shown in Figure 7D, IFN-γ suppressed the IL-1β regulation of MMP-3 but did not alter TIMP-1 expression. Similar data were also observed for MMP-1 and MMP-9, and gelatin zymography showed IFN-γ to inhibit the enzymatic activity of MMP-2 and MMP-9 (Figure S7ii). Second, transfection studies with a plasmid encoding a constitutively active form of STAT1 (STAT1-C) downregulated both basal and IL-1β-induced MMP-3 production by HPMCs (Figure 7E). To underscore a relationship between recurrent infections, MMP activity, and tissue injury, renal patients on stable PD were divided according to their prior history of bacterial peritonitis. Analysis of MMP3 and TIMP-1 levels in these cohorts showed an alteration in the balance between MMP and TIMP expression. Although there was no substantial difference in absolute MMP-3 and TIMP-1 levels in this patient group, a comparison of median values showed a marked decrease in MMP-3 in patients with prior infection history (Figure S7ii). When standardized against TIMP-1 levels, this reflected a significant decrease in the MMP-3/TIMP-1 ratio, which would predict increased matrix deposition and peritoneal fibrosis. Thus, IFN-γ regulates peritoneal MMP activities, which may reflect clinical changes in the homeostatic turnover of excellular matrix seen in peritoneal dialysis.

## Discussion

Studies examined the cellular events that dictate transition of an acute resolving inflammatory response into a more damaging inflammatory setting. Peritonitis provides an excellent opportunity to evaluate this process, because recurrent infections in renal failure patients on peritoneal dialysis is a major reason for treatment failure. Here, the frequency or severity of peritonitis correlates with increased tissue fibrosis, vascular damage, and the retention of activated leukocytes in the peritoneal cavity (Davies et al., 1996, Williams et al., 2002). To understand how recurrent peritonitis drives tissue injury, we developed a murine model of peritoneal inflammation, which promotes peritoneal fibrosis after repeated challenge with a microbial-derived stimulus (SES). This approach allowed us to track temporal changes in the inflammatory response that affect peritoneal cytokine production, leukocyte recruitment and activation, and the response of the resident stromal compartment.

Acute inflammation is a rapidly resolving process, which is self-limiting to restrict the development of tissue damage. We now show that recurrent acute episodes distort this process to cause fibrosis. This response was totally IL-6 dependent, and repeatedly challenged *Il6*^−/−^ mice displayed no histological evidence of peritoneal fibrosis. Fibrosis onset was not, however, linked to traditional profibrotic cytokines, and *Smad3*^*−/−*^ mice showed comparable pathology to WT mice. Instead, IL-6 promoted a robust Th1 cell-mediated response that disrupted the normal turnover of extracellular matrix through enhanced STAT1 signaling within the stromal compartment. Increased STAT1 activity was observed only in tissue that had encountered multiple rounds of acute inflammation and *Ifng*^*−/−*^, *Stat1*^*−/−*^, and *Rag1*^*−/−*^ mice were all protected from pathology. These results support the role of IL-6 in memory recall and emphasize the importance of IFN-γ-producing Th1 cells in protective immunity against bacterial infection (Longhi et al., 2008, Mills et al., 1993). Thus, a memory response to SES would be anticipated to support antimicrobial immunity but inadvertently drives tissue injury through STAT1 inhibition of homeostatic extracellular matrix turnover.

Competent antimicrobial host defense relies on IL-6 activity, and *Il6*^−/−^ mice show a reduced capacity to clear both viral and bacterial infections (Kopf et al., 1994, Lee et al., 1999, Longhi et al., 2008, Murphy et al., 2008). IL-6 activation of STAT3 controls neutrophil clearance and promotes T cell recruitment during acute peritoneal inflammation (Fielding et al., 2008, McLoughlin et al., 2005). Here, IL-6 and STAT3 signaling contributes to the control of antimicrobial immunity by regulating various innate immune activities to acute resolving or lethal infection (Greenhill et al., 2011, Kano et al., 2003, Matsukawa et al., 2003). For example, patients with hyper-IgE syndrome resulting from mutations in *STAT3* have impaired IL-6 signaling and suffer recurrent infections (Holland et al., 2007, Minegishi et al., 2007). A similar susceptibility to infection is also seen in individuals with clinically relevant IL-6 autoantibodies (Puel et al., 2008). Whereas STAT3 is heavily linked with chronic disease progression and cancer, this is often influenced by a signaling crosstalk with some other transcriptional regulators (Bollrath et al., 2009, Grivennikov et al., 2009, Jenkins et al., 2005, Jenkins et al., 2007, Judd et al., 2006, Nowell et al., 2009). Thus, the protective activities of IL-6 must be balanced against its ability to drive deleterious tissue injury. Our studies show no substantial increase in STAT3 activity between peritoneal tissue extracts taken from the first and fourth episode of acute inflammation. Instead, STAT3 activity in the fourth SES episode was accompanied by increased STAT1 signaling, which promoted fibrosis.

The relationship between IL-6 and IFN-γ is striking given the often distinct roles of STAT1 and STAT3 in inflammation and cancer. Increased local IFN-γ expression after repeat inflammatory activation would provide one potential mechanism by which the STAT1 versus STAT3 balance is altered to dictate disease outcome. This is akin to the control of STAT signaling in liver injury where tissue damage is associated with hyperactivation of STAT1 and reduced STAT3 activity (Hong et al., 2002, Ogata et al., 2006). The involvement of IFN-γ in tissue remodeling and disease progression is further emphasized by observations in double *Tcra*^*−/−*^*Socs1*^*−/−*^ mice where colitis is characterized by excessive IFN-γ-mediated STAT1 signaling (Chinen et al., 2006). Studies show that IFN-γ and STAT1 affects the turnover of extracellular matrix and can block fibrinolytic processes (Ho et al., 2008, Hu et al., 2005). However, other models highlight conflicting roles for IFN-γ in fibrosis. For example, IFN-γ has been described as an antifibrotic regulator that controls collagen synthesis and deposition (Gurujeyalakshmi and Giri, 1995, Oldroyd et al., 1999, Wynn et al., 1995). Importantly, cytokines including IL-4, IL-5, and IL-13 in association with TGF-β drive collagen production and promote fibrotic extracellular matrix remodeling (Wynn, 2004). This model of tissue damage is likely to apply to inflammatory situations where robust Th2 cytokine responses are implicated. Our data showed that an absence of T cell IL-6Rα signaling resulted in the SES-mediated expansion of IL-4- and IL-13-secreting T cells. Whereas these findings support a role for IL-6 in Th1 cell differentiation over that of Th2 cells, they also emphasize that T cell-derived IL-4 and IL-13 are not responsible for the lack of pathology seen in *Il6*^−/−^ mice. Our T cell studies show that SES control of IL-6 was essential for the survival and maintenance of T cell effector functions. This is consistent with the role of IL-6 in T cell recruitment, activation, and survival. Interleukin-6 governs the effector characteristics of various T cells subsets including Th17 cells, Th22 cells, and certain IL-10-secreting subsets (Stumhofer et al., 2007). Here, the nature of the T cell response may be influenced by innate sensing mechanisms. Thus, the pattern of cytokine expression displayed in response to an allergen may be distinct from that activated by a Gram-positive bacteria and is designed to steer a unique set of effector functions selected to combat the type of pathogen encountered. In this context, SES activates Th1 cell expansion in a IL-6Rα-dependent manner, which promotes enhanced stromal STAT1 signaling as an early prerequisite to the onset of peritoneal fibrosis.

The data presented here document a potential mechanism to explain how repeat acute resolving inflammation or infection drives tissue damage. Here, recurrent innate inflammatory activation inadvertently promotes adaptive immune responses that alter the pattern of cytokine signaling in stromal tissue, which ultimately gives rise to fibrosis and chronicity.

## Experimental Procedures

### Mouse Strains

All procedures were performed under Home Office project licenses 30/2269 and 30/2938 or Monash Medical Centre ‘A’ Animal Ethics approval. Inbred wild-type (WT) C57BL/6 mice were purchased from Charles River UK. IL-6-deficient (*Il6*^−/−^) (Kopf et al., 1994), IL-6R-deficient (*Cd126*^*−/−*^) (Jones et al., 2010), *Smad3*^*−/−*^ (Zhu et al., 1998), and IFN-γ-deficient (*Ifng*^*−/−*^) mice, recombinase-activated gene-1 (*Rag1*^*−/−*^) on a C57BL/6 background were bred in house from breeding pairs originally purchased from The Jackson Laboratory or obtained from GlaxoSmithKline (*Cd126*^*−/−*^). *Stat1*^*−/−*^ and WT littermates on a 129/C57BL/6 background were bred in house (from I. Campbell, University of Sydney, Australia). All mice were aged between 8 and 12 weeks and were weight matched for each experiment.

### Preparation of SES and Induction of Repeated Acute Inflammation

*Staphylococcus epidermidis* cell-free supernatant (SES) was prepared as previously described (Hurst et al., 2001, McLoughlin et al., 2003). Peritoneal inflammation was induced in WT and genetically modified mice through administration (i.p.) of SES (Hurst et al., 2001, McLoughlin et al., 2003). Mice were repeatedly challenged with four sequential episodes (7 days apart) of SES and mice were maintained for a maximum of 49 days before sacrifice. At defined intervals, the composition of the leukocyte infiltrate was assessed by a Coulter counter (Coulter Z2, Beckman Coulter), differential cell counting, and multiparameter flow cytometry. Peritoneal tissue and lavage fluids were harvested for biochemical analysis (see Supplemental Experimental Procedures). For inhibition of MMP activity, WT and *Il6*^−/−^ were repeatedly injected with SES as described. At day 43, day 45, and day 47, *Il6*^−/−^ mice received drinking water containing a collagenase-specific MMP inhibitor (Ro32-355 at dose of 12.5 mg/50 ml calculated to give a daily dose of 50 mg/kg). Groups of WT and *Il6*^−/−^ mice received the ethanol vehicle alone. Peritoneal membranes were harvested at day 49.

### Peritoneal Membrane Histopathology

Human parietal peritoneum sections were prepared as previously described (Williams et al., 2002). Murine parietal peritoneum (1 cm^2^) was harvested and sectioned in a similar manner. Biopsies were fixed with neutral buffered formal saline for 24 hr at 4°C and embedding in paraffin. Serial sections (of 5 μm thickness) were stained with hematoxylin and eosin. The thickness of the submesothelial cell compact zone was measured at three points over six fields of view along the length of the peritoneal section where an intact surface mesothelial layer was visible with the ×40 objective. Sections were immunostained with a specific rabbit anti-mouse collagen type-1 polyclonal antibody (2150-1410; AbD Serotec, MorphoSys UK) after antigen retrieval by digestion with trypsin. Staining was visualized with an anti-rabbit ABC detection kit and DAB (Dako) and counterstained with hematoxylin in a Dako autostainer. Slides were analyzed with a Leica DFC49 microscope and camera (Leica).

### SES-Driven Differentiation of T Helper Cells

Cells were cultured in RPMI 1640 supplemented with 10% (v/v) heat-inactivated FCS, 2 mM L-glutamine, 1 mM sodium pyruvate, 100 U/ml penicillin, 100 μg/ml streptomycin, and 55 mM 2-mercaptoethanol (all from Life Technologies). SES was reconstituted in 1 ml supplemented RPMI 1640 medium. Conditioned media from SES-stimulated peritoneal cells (SES-CM) was prepared by adding SES (1:1) to 1 × 10^6^ cells in a 1 ml final culture volume. FACS-sorted naive CD4^+^CD25^−^CD44^lo^CD62L^hi^ cells were cultured in 96-well plates at 1 × 10^5^ cells/well and stimulated with plate-bound anti-CD3 (1 μg/ml; 45-2C11) and soluble anti-CD28 (5 μg/ml; 37.51). Cultures were supplemented with SES-CM (1:1) or recombinant IL-12 (20 ng/ml) and cultured for 4 days. For the final 4 hr of culture, cells were restimulated with 50 ng/ml PMA and 500 ng/ml ionomycin in the presence of 3 μM monensin. Th1, Th2, and Th17 cell lineage differentiation was assessed by flow cytometry, by means of antibodies to CD4 (RM4-5), IFN-γ (XMG1.2), IL-4 (11B11), IL-6 (MP5-20F3), IL-9 (RM9A4), IL-13 (eBio13A), and IL-17 (TC11-18H10.1).

### Adoptive Transfer of Th1 Cells

Naive CD4^+^ T cells were expanded into Th1 cells in vitro with SES-CM. The proportion of IFN-γ^+^CD4^+^ T cells was determined by flow cytometry and used to calculate the number of cells for transfer into *Il6*^−/−^ or *Cd126*^*−/−*^ mice. Cells (0.5–1.0 × 10^6^) were coadministered to mice with SES (i.p.). Control mice received an equivalent number of sorted naive (Th0) CD4^+^ T cells. For quantification of peritoneal IFN-γ and immunoblot of STAT1 activity, cells were coadministered with a single dose of SES (a 3 hr stimulation). For evaluation of peritoneal fibrosis, cells were coadministered to mice with SES on day 0, 7, 14, and 21. Peritoneal fibrosis was assessed on day 49 as outlined above.

### Primary Human Mesothelial Cell Cultures

Primary human mesothelial cells (HPMCs) were isolated by tryptic digest from omental biopsies and cultured as previously described (Hurst et al., 2001, McLoughlin et al., 2003). Growth-arrested HPMCs were stimulated with 100 pg/ml IL-1β (R&D Systems) with or without 100 U/ml IFN-γ (Peprotech) for 72 hr and cell-free culture supernatants were prepared. A 60%–80% confluent HPMCs monolayer in 24-well plates was transfected with Xfect transfection reagent (Clontech) and 0.5 μg plasmid DNA (pcDNA3 vector control or pcDNA3 containing cDNA encoding a constitutively active STAT1 mutant, STAT1-C) per well for 3 hr in complete M199 medium containing 10% (v:v) FCS. After 24 hr, the media was replaced with fresh complete M199 medium containing 10% (v:v) FCS with or without IL-1β (100 pg/ml) and culture supernatants and protein lysates were prepared 24 hr later.

### Statistics

Student’s t test or Mann-Whitney tests in the GraphPad Prism software assessed statistical significance (GraphPad). p ≤ 0.05 was considered significantly different.

## Supplemental Information

Document S1. Supplemental Experimental Procedures and Figures S1–S7
