# Supplementary material for: Interleukin-6 Signaling Drives Fibrosis in Unresolved Inflammation
Source: Immunity. 2014 Jan 16;40(1):40–50. doi: 10.1016/j.immuni.2013.10.022 (PMC3919204; doi:10.1016/j.immuni.2013.10.022)
Supplement: Document S1. Supplemental Experimental Procedures and Figures S1–S7 [file mmc1.pdf]

Immunity, Volume *40*

Supplemental Information

Interleukin-6 Signaling Drives Fibrosis in Unresolved Inflammation

Ceri A. Fielding, Gareth W. Jones, Rachel M. McLoughlin, Louise McLeod, Victoria J. Hammond, Javier Uceda, Anwen S. Williams, Mark Lambie, Thomas L. Foster, Chia-Te Liao, Christopher M. Rice, Claire J. Greenhill, Chantal S. Colmont, Emily Hams, Barbara Coles, Ann Kift-Morgan, Zarabeth Newton, Katherine J. Craig, John D. Williams, Geraint T. Williams, Simon J. Davies, Ian R. Humphreys, Valerie B. O'Donnell, Philip R. Taylor, Brendan J. Jenkins, Nicholas Topley, and Simon A. Jones

Inventory of Supplemental information

Figure S1 (related to Figure 1)
SES-induced peritoneal fibrosis is IL-6 dependent, but is independent of classical pro-fibrotic cytokines.

Figure S2 (related to Figure 1)
SES-induced peritoneal fibrosis in IL-10KO mice is comparable to that seen in WT mice.

Figure S3 (related to Figure 1)
Evaluation of classical pro-fibrotic cytokines in SES-induced peritoneal inflammation.

Figure S4 (realted to Figure 2)
Densitometry analysis of NFκB and STAT EMSA results from WT and Il6$^{-/-}$ mice.

Figure S5 (related to Figure 3)
An increase in peritoneal IFN-γ producing CD4^{+} T-cells *is associated with fibrosis.*

Figure S6i (related to Figure 4)
Detection of Anti-SES specific IgG in peritoneal lavage fluid and analysis of the antigenicity of SES.
Figure S6ii (related to Figure 5)
IL-12p40 production by peritoneal DC, and IL-6 control of IL-12-driven Th1 cell expansion.

Figure S7i (related to Figure 6)
STAT1 and IFN-γ *producing Th1 cells contribute to peritoneal fibrosis .*
Figure S7ii (related to Figure 7)
IFN-γ-STAT1 regulates the MMP:TIMP balance and the development of fibrosis.

Supplemental Experimental Procedures-

1. Measurement of IgG specific for SES-
2. Analysis of nuclear extracts
3. Immunoblot analysis of peritoneal biopsies
4. Analysis of SES by SDS PAGE-
5. Quantitative real-time PCR (qPCR) of gene expression within the peritoneal membrane
6. Immunodetection of inflammatory mediators

7. Intracellular cytokine staining
8. SES-driven innate B-cell responses
9. Analysis of cytokine production by peritoneal DC-
10. Analysis of resident F4/80^{hi}CD11b^{hi} macrophage cytokine production by luminex cytokine profiling-
11. Analysis of MMP-2 and MMP-9 activity by zymography-

<u>Supplemental References-</u>

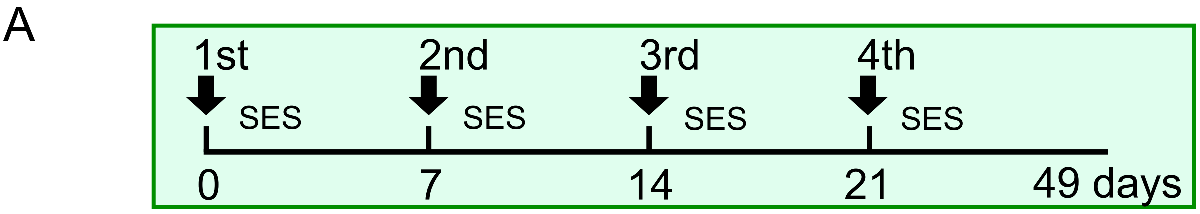

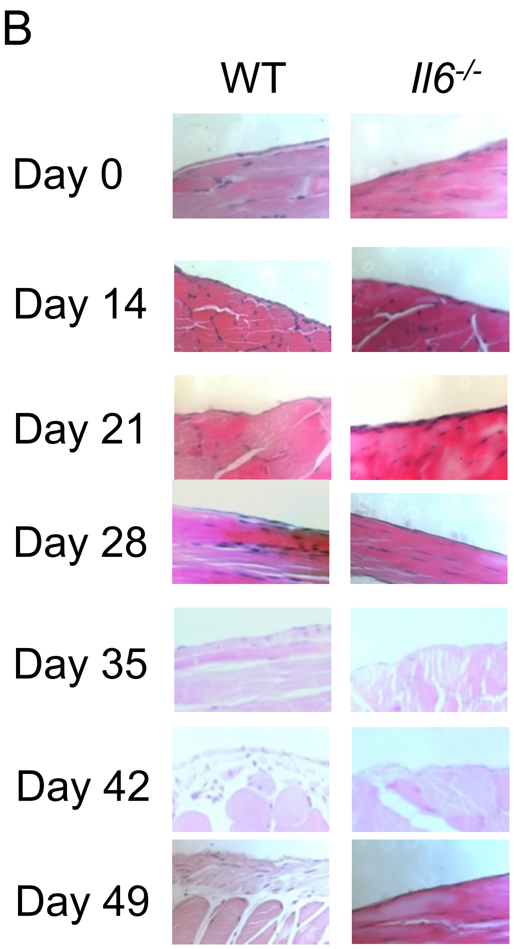

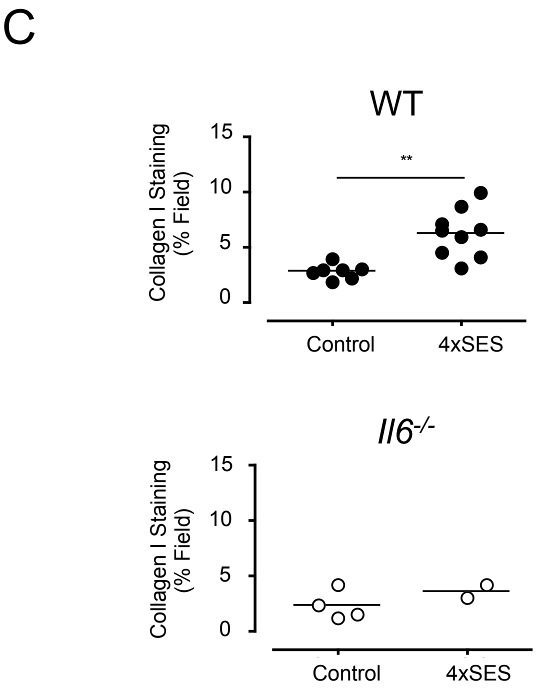

	Control (Day 49)	4xSES (Day 49)
WT	10.0±0.7μm	20.9±1.5μm***
Il6$^{-/-}$	6.6±0.6μm	6.7±1.0μm

*** $P \leq 0.001$ WT 4xSES vs WT Control and *Il6*$^{-/-}$ 4xSES

Figure S1

Figure S1. SES-induced peritoneal fibrosis is IL-6 dependent, but is independent of classical pro-fibrotic cytokines.

(A) Protocol description of the repeat SES-induced inflammation. Mice received 4 consecutive doses of SES (i.p.) administered at 7-day intervals. Histological evaluation of peritoneal fibrosis was recorded on day-49 post first SES challenge. (B) Sections of peritoneal membrane (5μm) were taken from SES-treated and age-matched control mice between Day 0 and Day 49 and stained with haematoxylin/eosin (H+E) and examined for thickening of the sub-mesothelial compact zone (layer between the muscle and membrane surface). Representative fields are shown from two individual mice per group (x400 magnification). Scale bar, 50μm. (C) Immunohistochemistry staining for Type I Collagen was quantified using the QWin software and adjusted for the length of peritoneal membrane within each field. Representative H&E histology sections (x400 magnification; scale bar, 50μm) are also shown. The table highlights the thickeness of peritoneal membranes at baseline and following SES challenge for WT and *Il6*$^{-/-}$ mice.

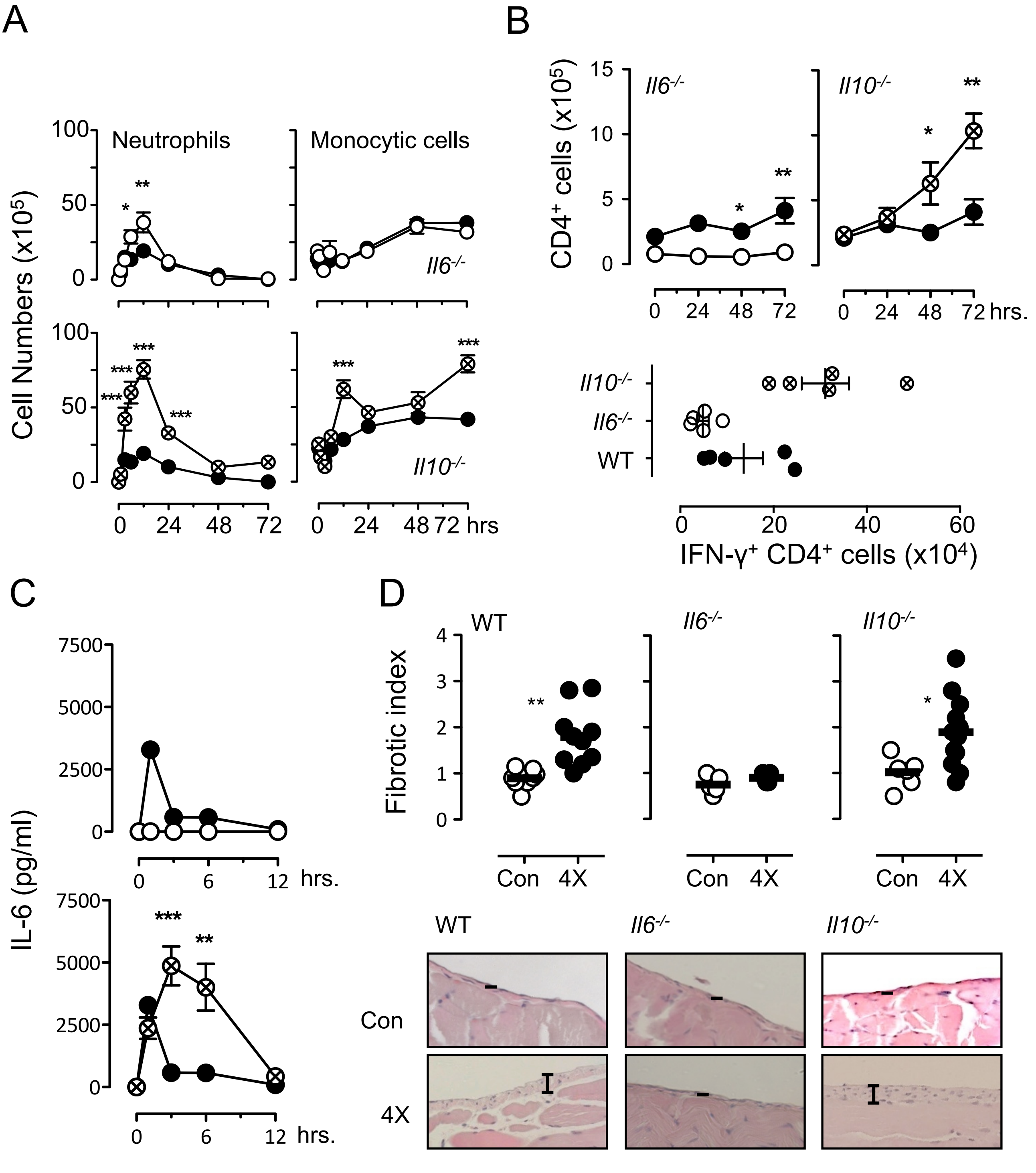
A
Neutrophils
Monocytic cells
Cell Numbers (x10^5)
Il6-/-
Il10-/-
0 24 48 72 hrs
B
CD4+ cells (x10^5)
Il6-/-
Il10-/-
0 24 48 72 hrs.
WT
IFN-γ+ CD4+ cells (x10^4)
C
IL-6 (pg/ml)
0 6 12 hrs.
D
Fibrotic index
WT
Con 4X
Con
4X

Figure S2

Figure S2. SES-induced peritoneal fibrosis in IL-10KO mice is comparable to that seen in WT mice.

(A-D) Increased SES-driven inflammation in *Il10*$^{-/-}$ mice does not equate to exacerbated peritoneal fibrosis. WT (closed circles), *Il6*$^{-/-}$ (open circles) and *Il10*$^{-/-}$ (crossed circles) mice were challenged (i.p.) with SES. At defined intervals the peritoneal cavity was lavaged. (A) Neutrophil and monocytic cell numbers were compared by differential cell counting. Data are represented as mean ± SEM. (B) Flow cytometric analysis of infiltrating CD4^{+} T-cells (upper panel). Numbers of infiltrating IFNγ-secreting CD4^{+} T-cells were monitored by intracellular flow cytometry using cells isolated 72 hours post SES challenge (lower panel). Data are represented as mean ± SEM. (C) ELISA quantification of IL-6 in peritoneal lavage from SES challenged mice *Il6*$^{-/-}$ (upper panel) and *Il10*$^{-/-}$ (lower panel) mice. The mean ± SEM (n=5-15 mice per time point, *P<0.05, **P<0.01, ***P<0.001) is presented. (D) Mice were challenged with 4 consecutive rounds of SES-induced peritoneal inflammation. Thickening of the sub-mesothelial compact zone was scored as a fold-change from non-stimulated WT controls at Day-49. Data from control mice and mice receiving 4 bouts of SES-induced inflammation (4X) are presented (n=5-10 mice per test condition, *P<0.05, **P<0.01). Representative H&E stained 5μm sections (x400 magnification) are shown where the bars illustrate the thickness of the sub-mesothelial compact zone.

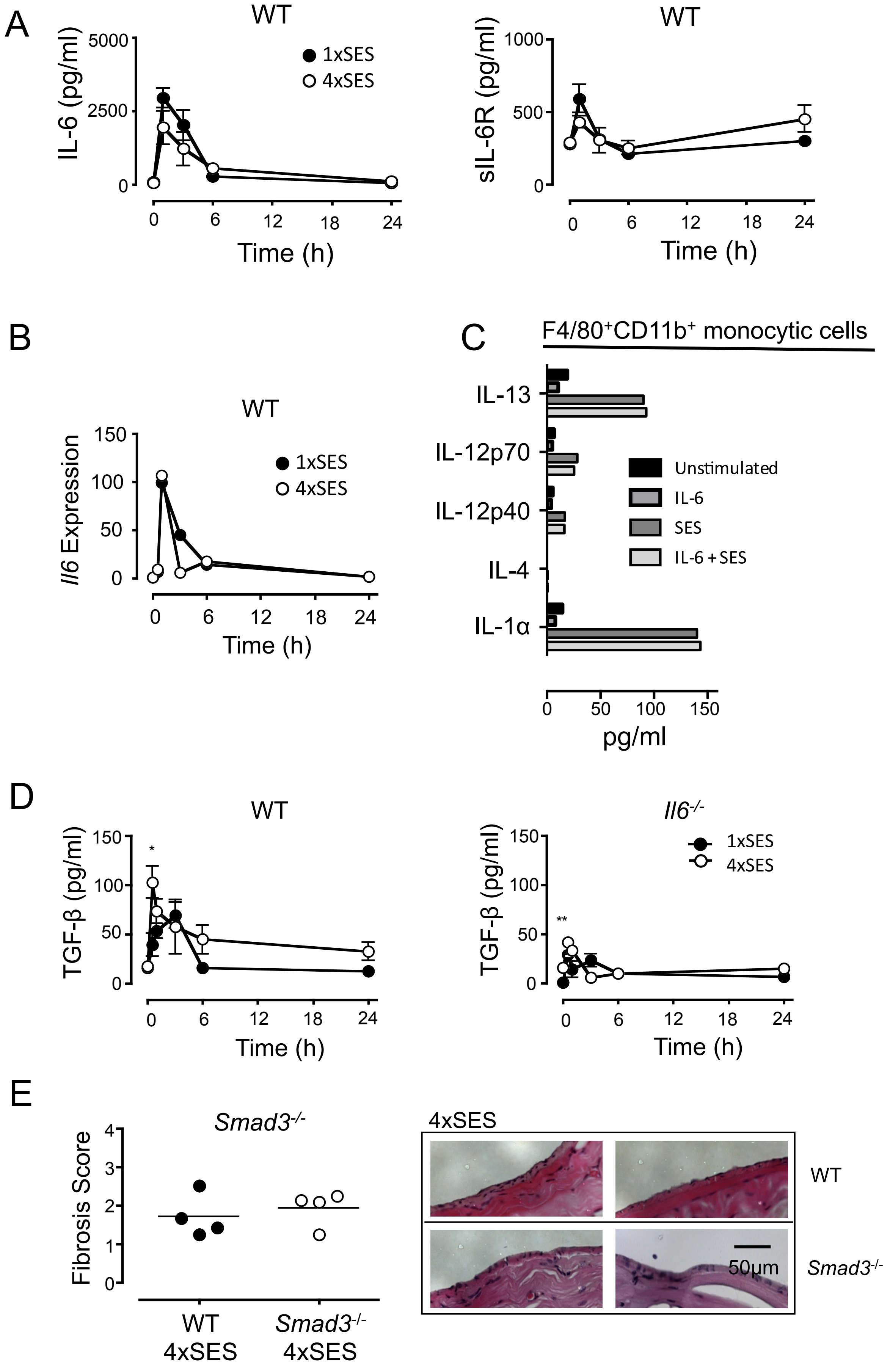
A
WT
IL-6 (pg/ml)
1xSES
4xSES
Time (h)
WT
sIL-6R (pg/ml)
Time (h)
B
WT
Il6 Expression
1xSES
4xSES
Time (h)
C
F4/80+CD11b+ monocytic cells
IL-13
IL-12p70
IL-12p40
IL-4
IL-1α
Unstimulated
IL-6
SES
IL-6 + SES
pg/ml
D
WT
TGF-β (pg/ml)
Time (h)
Il6-/-
1xSES
4xSES
TGF-β (pg/ml)
Time (h)
E
Smad3-/-
Fibrosis Score
WT 4xSES
Smad3-/- 4xSES
4xSES
WT
Smad3-/-
50µm

Figure S3

Figure S3. Evaluation of classical pro-fibrotic cytokines in SES-induced peritoneal inflammation.
(A) IL-6 and sIL-6R within peritoneal lavage fluid were measured by ELISA in WT mice. Results shown are the mean ± SEM (*n*≥5 per time point). (B) Q-PCR for *Il6* expression in mRNA from peritoneal membranes of SES challenged WT mice. (C) Resident F4/80^{hi}CD11b^{hi} macrophages were sorted from pooled peritoneal exudates. A luminex screen of cytokine production by F4/80^{+}CD11b^{+} cells was performed in response to medium, SES, IL-6 (10ng/ml) or a combination of SES and IL-6. Data is from a single experiment performed in duplicate. (D) TGF-β production (mean ± SEM; *n*≥5 per time point) was measured in WT and *Il6*$^{-/-}$ mice by specific ELISA within peritoneal lavage fluid from episodes 1 (1xSES) and 4 (4xSES). (E) Sections of peritoneal membrane (5μm) taken from SES-treated WT and *Smad3*$^{-/-}$ mice on Day 49 were stained with haematoxylin/eosin and examined for thickening of the sub-mesothelial compact zone (layer between the muscle and membrane surface). Representative fields are shown from two individual mice per group (x400 magnification; Scale bar, 50μM). Fibrotic scores for SES challenged WT and *Smad3*$^{-/-}$ mice are shown (n=4 per group, no significant difference observed). Scores reflect a fold-change in sub-mesothelial zone thickness compared to WT controls at Day 49. Related to Figure 1.

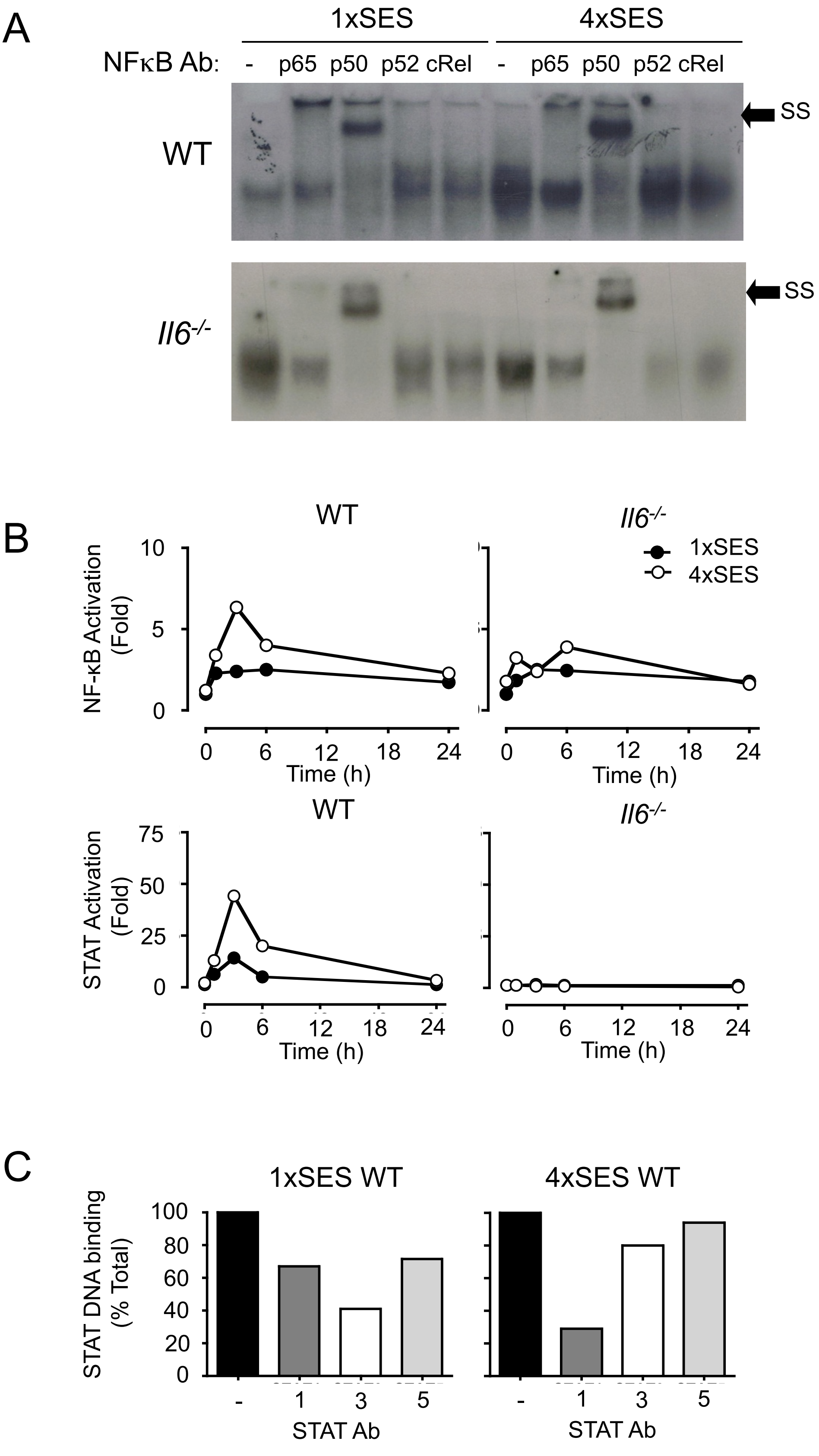
A
1xSES
4xSES
NFκB Ab:
- p65 p50 p52 cRel
- p65 p50 p52 cRel
WT
Il6-/-
SS
SS
B
WT
Il6-/-
1xSES
4xSES
NF-κB Activation (Fold)
Time (h)
STAT Activation (Fold)
Time (h)
C
1xSES WT
4xSES WT
STAT DNA binding (% Total)
STAT Ab
STAT Ab

Figure S4

Figure S4. Densitometry analysis of NFκB and STAT EMSA results from WT and Il6$^{-/-}$ mice.

(A) The composition of the NFκB DNA-binding complex in WT and *Il6$^{-/-}$* mice was analyzed by addition of antibodies specific for the different NFκB family members (p65, p50, p52 and c-Rel), using the peak timepoint (3hr sample) from the first and fourth episodes. Results shown are representative of nuclear extracts prepared from 3 different mice per time point and genotype. Antibody-induced NFκB supershift (SS) is indicated by black filled arrows. (B) The changes in NFκB and STAT activation observed by EMSA (Figure 2A) were quantified by densitometry using the Image J software and the results expressed as fold-change over basal DNA-binding, following correction using the NF1 probe. Representative results obtained from a single EMSA are shown. (C) Densitometric analysis was also applied to STAT supershift EMSA (see also Figure 2B) and the results expressed as a % of the STAT DNA-binding observed in the absence of antibody. Related to Figure 2.

A

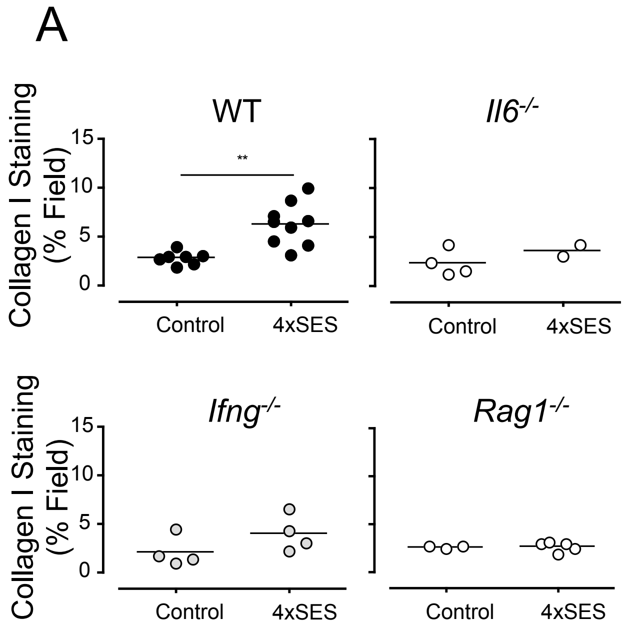

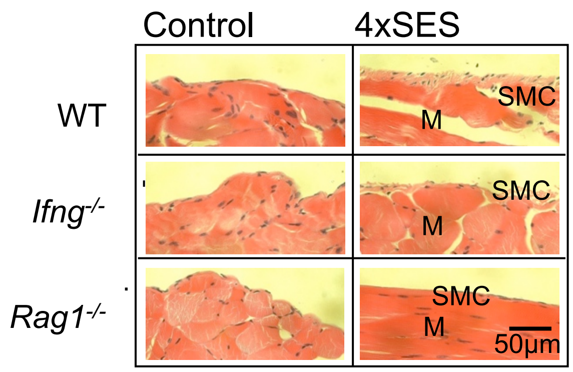

	Control (Day 49)	4xSES (Day 49)
WT	10.0±0.7μm	20.9±1.5μm***
Il6$^{-/-}$	6.6±0.6μm	6.7±1.0μm
Ifng$^{-/-}$	15.7±1.0μm	16.0±2.7μm
Rag1$^{-/-}$	9.1±0.7μm	12.9±3μm

*** $P \leq 0.001$ WT 4xSES vs WT Control and *Il6*$^{-/-}$ 4xSES

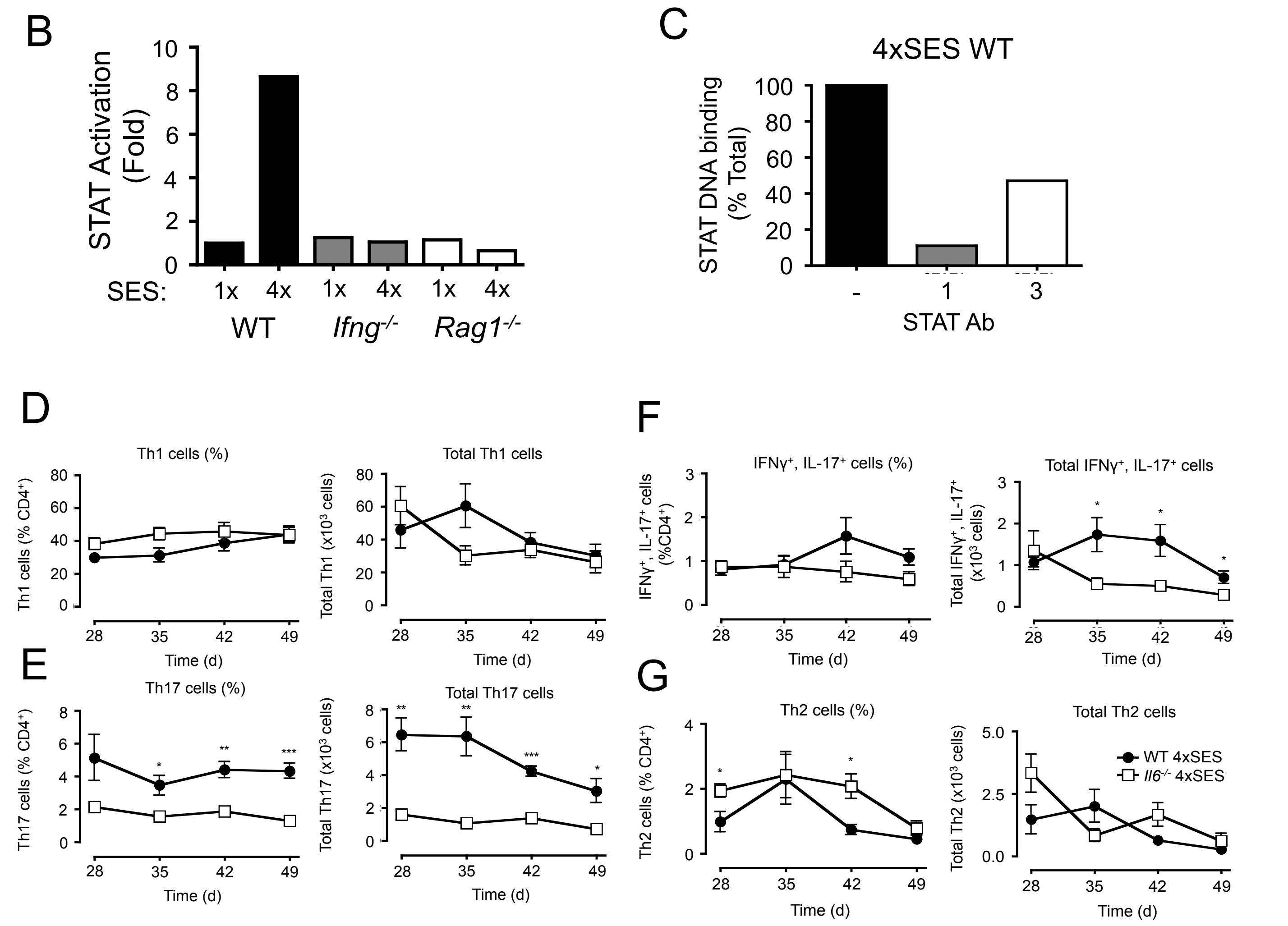

Figure S5

Figure S5. An increase in peritoneal IFN-γ producing CD4+ T-cells *is associated with fibrosis.*

(A) Immunohistochemistry staining for Type I Collagen was quantified using QWin software and adjusted for the length of peritoneal membrane within each field. The table highlights the thickeness of peritoneal membranes at baseline and following SES challenge in WT, *Il6*$^{-/-}$, *Ifng*$^{-/-}$ and *Rag*$^{-/-}$ mice. Representative histology sections (x400 magnification; scale bar, 50μm) are also shown. (B) Comparison of STAT activation observed by EMSA (see also Figure 3C) and quantified by densitometry using Image J software. Results are expressed as fold-change in DNA-binding from the WT 3hr time point during the first inflammatory episode, following, correction using the NF1 probe. (C) Densitometric analysis was also applied to STAT supershift EMSA (see Figure 3C) from the WT Episode 4 sample and the results expressed as a % of the STAT DNA-binding observed in the absence of antibody. (D-E) Peritoneal leukocytes were isolated from WT and *Il6*$^{-/-}$ mice by peritoneal lavage following the fourth inflammatory episode at Day 28 to Day 49. They were cultured in the presence of a Golgi blocker (monensin) and PMA/ionomycin for 4hrs at 37°C and then stained for cell surface CD4 prior to fixation and permeabilization and staining for intracellular IFN-γ and IL-17A. Peritoneal Th1 cells (D), Th17 cells (E), CD4+ cells secreting both IFN-γ and IL-17A (F), and Th2 cells (G) were expressed as a percentage of the CD4+ population and the actual number of cells within the peritoneal cavity. Data are represented as mean ± SEM. Related to Figure 3.

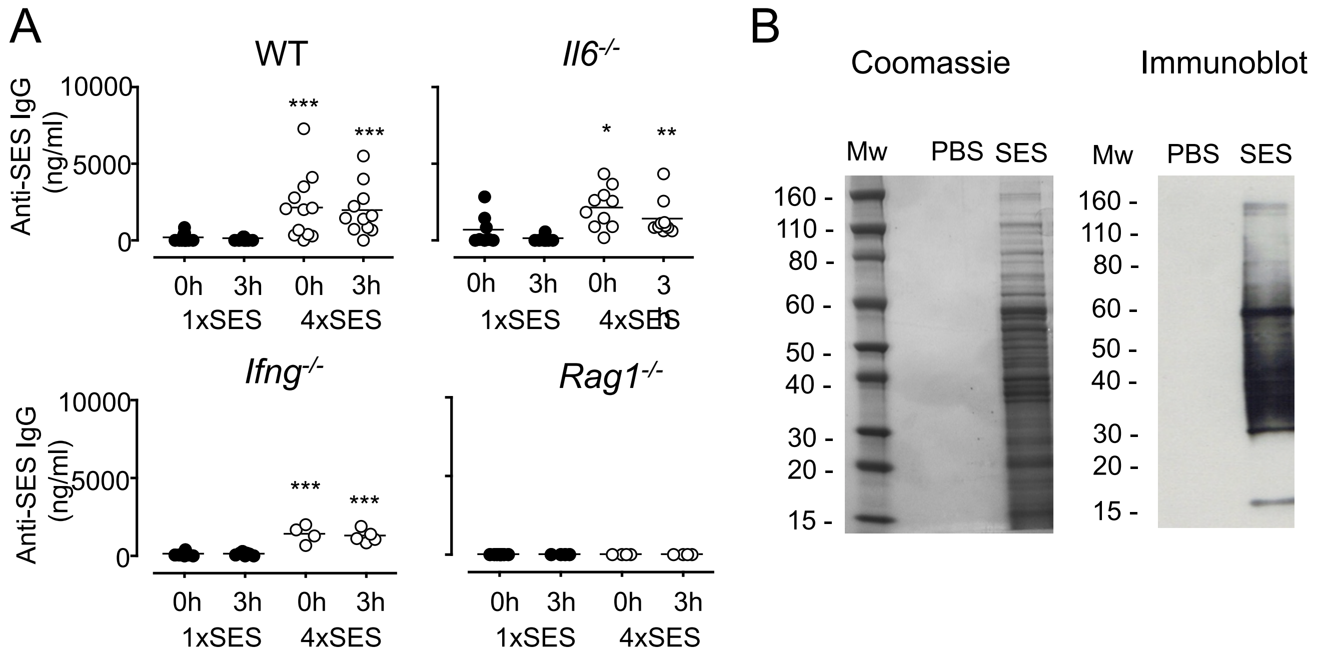

Figure S6i

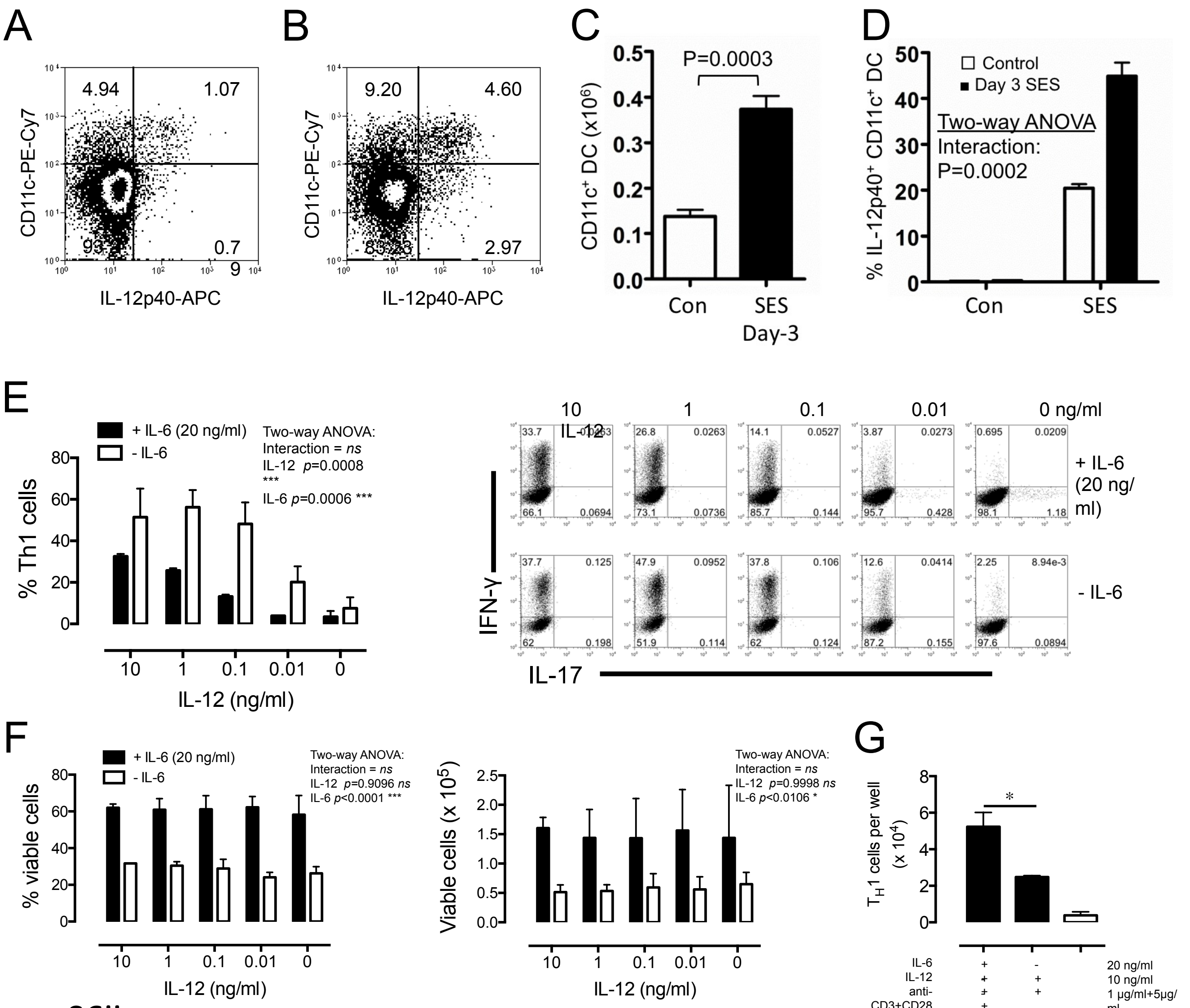

Figure S6ii

Figure S6i. Detection of Anti-SES specific IgG in peritoneal lavage fluid and analysis of the antigenicity of SES.

(A) The effect of repeated inflammation on antibody generation was examined by measurement of IgG specific for SES in lavage fluid from WT, *Il6*$^{-/-}$, *Ifng*$^{-/-}$ and *Rag1*$^{-/-}$ mice at 0 and 3 hours ($n \geq 5$ per group, unpaired t-test * $P<0.05$, **$P<0.01$, ***$P<0.001$ compared to Episode 1 for the respective time point and genotype). (B) SES was separated by SDS-PAGE on a 4-12% polyacrylamide gradient gel and detected by coomasie blue staining or immunostaining with a diluted peritoneal lavage fluid (WT Day 21 3hrs) as a primary antibody, followed by anti-mouse HRP secondary. Results are representative of at least two experiments. Related to Figure 4.

Figure S6ii. IL-12p40 production by peritoneal DC, and IL-6 control of IL-12-driven Th1 cell expansion.

Representative density plot of (A) naïve peritoneal cells stimulated with SES (10% v/v), and (B) peritoneal cells elicited 3 days after i.p. administration of SES . All cells were gated on F4/80^{+}CD11b^{+} cells after excluding eosinophils and doublets as described in the methods and CD19^{+}MHCII^{+} B cells. Plots are representative of 4 (A) and 5 (B) individual 6 week old C57BL/6 mice. (C) Quantification of the number of peritoneal DC in the mice represented in (A) and (B) above. Data shown represents the mean ± SEM and the difference was assessed with a Student's *t*-test. (D) The proportion of peritoneal DC producing IL-12p40 *ex vivo* was quantified and analysed by two-way ANOVA. The significant interaction statistic indicates DC from inflamed mice are more likely to be induced to prodice IL-12p40 after stimulation with SES. Data shown represents mean ± SEM of the mice represented in (A) and (B). (E) Th1 cell expansion under anti-CD3/anti-CD28 antibody co-stimulation in the presence of exogenous IL-6 (20ng/ml) and IL-12 (0.01-10ng/ml). The proportion of IFN-γ and IL-17A secreting CD4 T-cells was determined by intracellular flow cytometry following stimulation with PMA and ionomycin in the presence of monensin for 4 h prior. Data are representative of two independent experiments. (F) The viability of T-cells cultures was determined using the Muse Count and Viability Assay and Muse Cell Analyzer and is presented as a % of the total cell population and as an absolute CD4 T-cell number. (G) Quantification of Th1 cell numbers in cultures treated with exogenous IL-12 (10ng/ml) in the presence or absence of IL-6 (20ng/ml). Data are representative of two independent experiments (mean ± SEM; *$P<0.05$). Related to Figure 5.

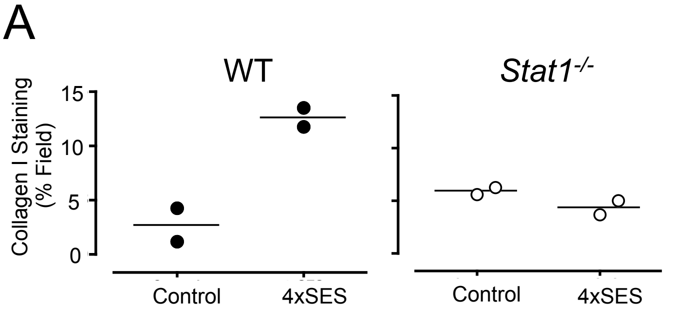

	Control (Day 49)	4xSES (Day 49)
WT (129/B6)	7.5±0.6μm	20.9±2.2μm**
Stat1$^{-/-}$	11.7±0.6μm	13.1±2.5μm

** *P*≤0.01 vs WT Control (Day 49)

** *P*≤0.01 WT and *Stat1*$^{-/-}$ Blank

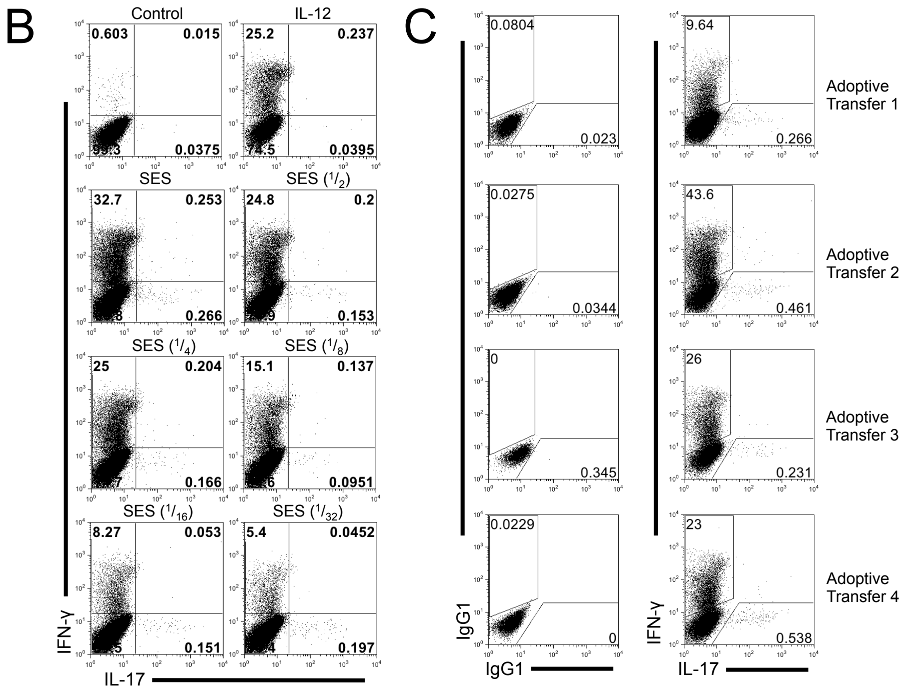

Figure S7i

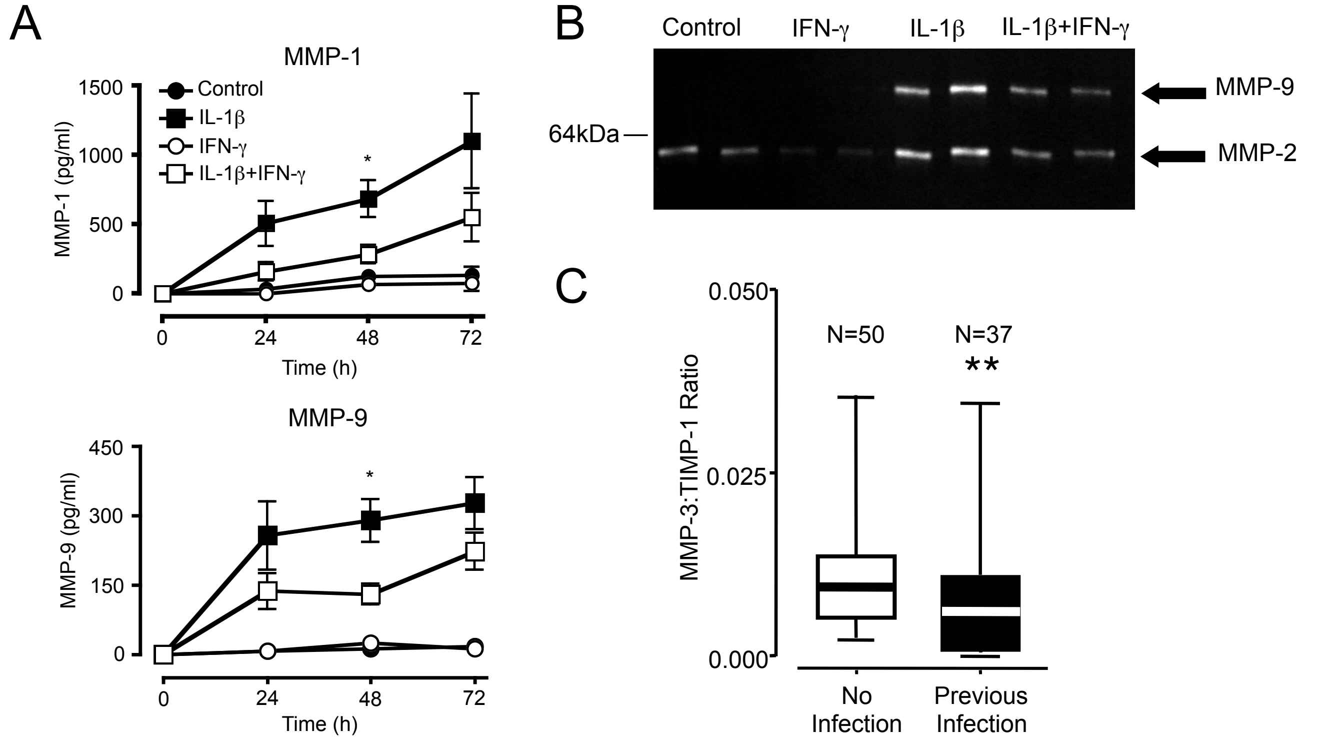

Figure S7ii

Figure S7i. STAT1 and IFN-γ *producing Th1 cells contribute to peritoneal fibrosis .*

(A) Evaluation of peritoneal fibrosis in WT and *Stat1*$^{-/-}$ mice. Sections of peritoneal membrane (5µm) were taken from *Stat1*$^{-/-}$ and WT age-matched control mice receiving 4 challenges of SES and assessed for peritoneal fibrosis at day 49 by immunohistochemistry for Type I Collagen. Staining was quantified using QWin software and adjusted for the length of peritoneal membrane within each field. The table shows analysis of peritoneal membrane thickness at baseline and following SES challenge. (B) Optimisation of T-cell expansion studies with conditioned media from SES challenged mononuclear cells. Naïve CD4 T-cells were cultured for 4 days with anti-CD3/CD28 co-stimualtory antibodies in the presence of SES conditioned media (Various dilutions are presented). The response to exogenous IL-12 (10ng/ml) is used as a positive control. CD4 T-cells were evaluated for Th1 differentiation by intracellular flow cytometry for IFN-γ and IL-17A. (C) Peritoneal cells were stimulated with SES for 24hrs. Conditioned media was recovered and added to naïve T cells (1:1) activated with anti-CD3/anti-CD28 for 4 days. Th1 cells (0.5–1.0 x 10^6 cells, corrected for the proportion secreting IFN-γ) were administered to *Il6*$^{-/-}$ at the same time as SES-challenge (i.p.). Flow cytometry plots are shown for the T-cell populations transferred to *Il6*$^{-/-}$ mice during each round of SES-induced inflammation and accompany the data presented in Figure 6. Related to Figure 6.

Figure S7ii. IFN-γ-STAT1 regulates the MMP:TIMP balance and the development of fibrosis.

(A) Growth-arrested human peritoneal mesothelial cells (HPMC) were treated with medium alone (control), IL-1β (100pg/ml), IFN-γ (100U/ml) or IL-1β in combination with IFN-γ for up to 72hrs. Cell free supernatants were analyzed for MMP-1 or MMP-9 by specific ELISA. (B) Cell free supernatants from above were resolved on a 10% non-denaturing polyacrylamide gel containing % gelatin by SDS-PAGE. Gels were incubated overnight in zymography buffer and stained with coomassie blue. Result shown were representative of 3 experiments. (C) MMP-3 and TIMP-1 were measured in samples of peritoneal dialysis effluent from stable PD patients with either no previous infection history or those with a previous history of infection. The ratio of MMP-3 to TIMP-1 was calculated for each sample and found to be significantly different in patient with a history of prior peritonitis (Mann-Whitney analysis ** P=0.0013). Related to Figure 7.

Supplemental Experimental Procedures-

Measurement of IgG specific for SES-

Anti-SES IgG levels were detected using a direct ELISA method. Briefly, 96 well ELISA plates were coated with serial dilutions of mouse IgG as a standard curve (highest concentration 200ng/ml) or SES in PBS overnight at room temperature, blocked and incubated with 1% BSA/PBS (IgG-coated wells) or peritoneal lavage fluid diluted in 1% BSA/PBS (SES-coated wells). Bound IgG was detected using horseradish peroxidase-conjugated anti-mouse IgG (1:500; Santa Cruz Biotechnology, Heidelberg, Germany) and a SureBlue TMB microwell peroxidase substrate (Insight Biotechnology, Wembley, UK). Plates were washed three times in between all steps with PBS containing 0.05% Tween-20, except between the final substrate and stop solution steps.

Analysis of nuclear extracts

Nuclear extracts were prepared from HPMC using a rapid technique for the extraction of DNA binding proteins (Andrews and Faller, 1991). Briefly, the peritoneal membrane was harvested and snap-frozen in liquid nitrogen before grinding into a fine powder using a pestle and mortar. The powder was resuspended in ice-cold buffer A (10mm HEPES-KOH (pH 7.9), 1.5mM $MgCl_2$, 10mM KCl, containing proteinase inhibitors (diluted 1:1000, Sigma-Aldrich), 0.5mM DTT, 0.2mM PMSF, 1mM sodium orthovanadate and 50mM NaF) and incubated on ice for 30 mins. The nuclear pellet was collected by centrifugation, resuspended in buffer B (20mM HEPES-KOH (pH 7.9), 25% Glycerol 420mM NaCl, 1.5mM $MgCl_2$ containing proteinase inhibitors (diluted 1:1000, Sigma-Aldrich), 0.2mM EDTA, 0.3mM DTT, 0.2mM PMSF, 1mM sodium orthovanadate and 50mM NaF) and incubated on ice for 30 mins to allow for high salt extraction. Nuclear debris was removed by brief high-speed centrifugation (12,000g for 5mins seconds, 4°C) and the resulting supernatants (nuclear extract) collected. Protein concentrations were determined using the Bradford method. Electrophoretic mobility shift assays (EMSA) were performed as previously described with 10μg nuclear extract (McLoughlin et al., 2003). Oligonucleotides containing consensus motifs for NF-κB (5'-gaTCCATGGGGAATTCCCC-3' and 3'-AGGTACCCCTTAAGGGGag -5') and the SIE m67 (STAT) (5'-cgaCATTTCCCGTAAATCG-3' and 3'-GTAAAGGGCATTTAGCagc-5') were annealed for use in EMSA. These double-stranded fragments were labeled with [α^{32}P]-dTTP (GE Healthcare UK, Little Chalfont, UK) using the Klenow fragment of DNA polymerase I. The composition of protein/DNA complexes was determined by supershift assays using 2μg per reaction of a rabbit polyclonal antibodies specific for either NF-κB p50 (NLS, sc-114), NF-κB p65 (A, sc-109), NF-κB p52 (C-5, sc-7386), NF-κB c-Rel (N-466, sc-

272), STAT1 (M-22, sc-592) or STAT3 (C-20, sc-482) (all Santa Cruz Biotechnology, Heidelberg, Germany). Densitometry of EMSA results was performed using the Image J open access software, provided by W. S. Rasband, U.S. National Institutes of Health website, Bethesda, Maryland (http://rsb.info.nih.gov/ij/).

Immunoblot analysis of peritoneal biopsies

Protein extracts from frozen peritoneal biopsies were prepared using ice-cold lysis buffer, following which they were pre-cleared of cellular debris prior to separation by SDS-PAGE and immunoblotting with specific antibodies (Jenkins et al., 2005). Immuno-labeled proteins were detected using either the enhanced chemiluminescence (ECL) detection system (Amersham Biosciences) or Odyssey Infrared Imaging System (LI-COR, Lincoln, New England) with the appropriate secondary antibodies as per the manufacturer's instructions.

Analysis of SES by SDS PAGE-

50µl PBS or one aliquot of SES was resuspended in 50µl PBS and mixed with 15µl Nupage 4x gel sample buffer (Invitrogen), 6µl DTT (1M) and denatured at 95°C for 5mins. 20µl PBS or SES per lane was separated by SDS-PAGE using a 4-12% Nupage polyacrylamide gel (Invitrogen). Gels were either stained with Novex Colloidal Blue staining kit (Invitrogen), according to the manufacturer's instructions or transferred to nitrocelluose (Hybond ECL, GE Life Science) by semi-dry blotting. Immunoblotting was carried out with diluted peritoneal lavage from WT mice (Day 21 3h, dilute 1:500) and horseradish peroxidase-conjugated anti-mouse IgG (1:2000; Santa Cruz Biotechnology, Heidelberg, Germany), and detected with Super Signal West Pico chemiluminescent reagent (Thermo Fisher Scientific).

Quantitative real-time PCR (qPCR) of gene expression within the peritoneal membrane

Murine parietal peritoneal membrane samples were homogenized in Trireagent (Invitrogen, Paisley, UK) and total RNA prepared according to the manufacturer's instructions. RNA integrity was confirmed by agarose gel electrophoresis and quantified using a spectrophotometer (Nanodrop). Complimentary DNA (cDNA) was prepared using the Superscript III cDNA synthesis kit (Invitrogen, Paisley, UK) and used in quantitative qPCR reactions either with Taqman primer and probe sets and mastermix (Applied Biosystems, Foster City, California) or with standard oligonucleotide primers and a Syber Green mastermix (Invitrogen, Paisley, UK). Quantitative-PCR acquisition and analysis was performed with Sequence Detection System version 2.3 software (Applied Biosystems). Relative expression levels were calculated from the CT values

for the target and endogenous control genes (ΔCT), relative to the WT baseline control (ΔΔCT) set to 1.

Immunodetection of inflammatory mediators

Murine TGF-β, IL-6 and IFN-γ (BD OptEIA kits), and human MMP-1 (Merck Biosciences/Oncogene Research Products), MMP-3, MMP-9, and TIMP-1 were quantified using commercial ELISA kits (Duoset kits from R&D Systems). Murine sIL-6R levels were quantified against recombinant murine sIL-6R (1830-SR) using the matched antibody pair AF1830 and BAF1830 (all from R&D Systems, Abbingdon, UK) (Chalaris et al., 2007). Human IFN-γ and MMP-3 were measured in peritoneal dialysis effluent using a custom Bioplex luminex kit (for mIFN-γ, BioRad) and 3-plex MMP assay plate (Meso Scale Discovery, Gainsville, Florida) respectively.

Intracellular cytokine staining

Peritoneal leukocytes were harvested and stimulated with 50ng/ml PMA and 500ng/ml ionomycin for 4hrs at 37°C in complete RPMI 1640 containing 3μM monesin, a Golgi blocker (all from Sigma). Stimulated leukocytes were immunostained with APC-conjugated anti-mouse CD4 antibodies to detect surface CD4 expression, fixed and permeabilized using a Fix/Perm kit, and immunostained with FITC-conjugated anti-mouse IFN-γ antibodies to detect intracellular IFN-γ expression (all reagents from BD Biosciences, Oxford, UK). Immunostained cells were analyzed by flow cytometry using a Becton Dickinson FACScalibur and the FlowJo analysis software.

SES-driven innate B-cell responses

WT mice were challenged (i.p.) for 6 hours with SES. The peritoneal cavity was lavaged and the recovered leukocytes stimulated for a further 4 hours *ex vivo* with SES (1:1 in in RPMI 1640 containing 3 μM monensin). Intracellular flow cytometry using antibodies against IFN-γ (XMG1.2) was recorded in gated CD4 (RM4-5) and CD19 (1D3) lymphocytes.

Analysis of cytokine production by peritoneal DC-

Peritoneal cells were harvested and washed three times with complete RPMI medium (RPMI 1640 medium supplemented with 10% heat-inactivated fetal calf serum, 10 U/ml penicillin and 10 μg/ml streptomycin). 5 x 10^5 cells were then aliquoted into 48 well flat-bottomed plates in a final volume of 200 μl of complete RPMI medium in the presence of 0.1% v/v Golgi-plug (BD Biosciences). Cells were incubated in medium alone, or stimulated with 10% (v/v) SES at 37 °C,

5% CO_2 for 6 hours. The cells were carefully recovered using plastic cell lifters and transferred to 96 well U-bottomed plates. The plates were spun down at 350 *g* for 5 min to harvest the cells for further intracellular flow-cytometric cytokine staining. Intracellular flow-cytometry was performed according to the conventional protocols. In brief, cells were first fixed with 1% formaldehyde (in PBS) for 20 min and permeabilized with wash buffer containing 0.5% (w/v) saponin (Sigma). Cells were then incubated in 50 µl blocking buffer (PBS containing 5% v/v heat-inactivated rabbit serum, 0.5% w/v BSA, 5 mM EDTA, 2 mM NaN3, 4 µg/mL rat anti-mouse FcɣRII&III (2.4G2) for 30 min at 4°C. Fluorochrome-labeled antibodies in wash buffer (PBS containing 0.5% w/v BSA, 5 mM EDTA, 2 mM NaN_3) [CD19-V450 (clone 1D3, BD), CD11b-FITC (clone 5C6, AbD Serotec), IL-12p40-PE (clone C15.6, BD), F4/80-PE-TxR (clone BM8, Life Technologies), MHCII-PerCP-Cy5.5 (clone M5114, Biolegend) and CD11c-PE-Cy7 (clone HL3, BD)] were added to make a final volume of 100 µl. After 30 minutes incubation at 4°C, they were washed three times with wash buffer. Samples were then acquired on a 9 colour 3 laser CyAn ADP Analyser (Beckmann) and analysed using Summit software (Beckmann). Peritoneal DC were identified after first gating on single cells events (via FSC_{lin} vs FSC_{Area} analysis) and exclucing eosinophils on FSC vs SSC plots (as described in Davies et al., 2011). Subsequently, $CD19^{+}MHCII^{+}$ B cells were excluded and the F4/80+CD11b+ myeloid cells were selected for analysis of CD11c expression and IL-12p40 production. Thus peritoneal DC were defined as $CD11c^{+}MHCII^{high}F4/80^{int}CD11b^{+}$ cells. Data were analysed using GraphPad Prism.

Analysis of resident $F4/80^{hi}CD11b^{hi}$ macrophage cytokine production *by luminex cytokine profiling-*

Resident $F4/80^{hi}CD11b^{hi}$ macrophages were sorted from pooled peritoneal exudates and stimulated *in vitro* with medium, SES, IL-6 (10ng/ml) or a combination of SES and IL-6. Cell-free supernatants were prepared. A panel of 23 different murine cytokines/inflammatory mediators were measured by using a commercial Bio-Plex Mouse Cytokine assay kit (Bio-Rad).

Analysis of MMP-2 and MMP-9 activity by zymography-

20µl HPMC culture supernatants were separated by SDS-PAGE on a 10% non-denaturing polyacrylamide gel containing 1mg/ml gelatin. Following electrophoresis, the gel was washed with 2.5% Triton X-100 for 1h and incubated overnight at 37°C in 50mM Tris-Cl pH7.5, 10mM $CaCl_2$ and staınined with Coomassie Blue.

Supplemental References-

Andrews, N.C., and D.V. Faller. 1991. A rapid micropreparation technique for extraction of DNA-binding proteins from limiting numbers of mammalian cells. *Nucleic Acids Res* 19:2499.

Chalaris, A., B. Rabe, K. Paliga, H. Lange, T. Laskay, C.A. Fielding, S.A. Jones, S. Rose-John, and J. Scheller. 2007. Apoptosis is a natural stimulus of IL6R shedding and contributes to the proinflammatory trans-signaling function of neutrophils. *Blood* 110:1748-1755.

Jenkins, B.J., D. Grail, T. Nheu, M. Najdovska, B. Wang, P. Waring, M. Inglese, R.M. McLoughlin, S.A. Jones, N. Topley, H. Baumann, L.M. Judd, A.S. Giraud, A. Boussioutas, H.J. Zhu, and M. Ernst. 2005. Hyperactivation of Stat3 in gp130 mutant mice promotes gastric hyperproliferation and desensitizes TGF-beta signaling. *Nat Med* 11:845-852.

McLoughlin, R.M., J. Witowski, R.L. Robson, T.S. Wilkinson, S.M. Hurst, A.S. Williams, J.D. Williams, S. Rose-John, S.A. Jones, and N. Topley. 2003. Interplay between IFN-gamma and IL-6 signaling governs neutrophil trafficking and apoptosis during acute inflammation. *The Journal of clinical investigation* 112:598-607.
